# Monomeric Triphosphinoboranes: Intramolecular Lewis Acid–Base Interactions between Boron and Phosphorus Atoms

**DOI:** 10.1021/acs.inorgchem.1c03618

**Published:** 2022-02-28

**Authors:** Anna Ordyszewska, Natalia Szynkiewicz, Jarosław Chojnacki, Rafał Grubba

**Affiliations:** Department of Inorganic Chemistry, Faculty of Chemistry, Gdańsk University of Technology, 11/12 Gabriela Narutowicza Strasse, 80-233 Gdańsk, Poland

## Abstract

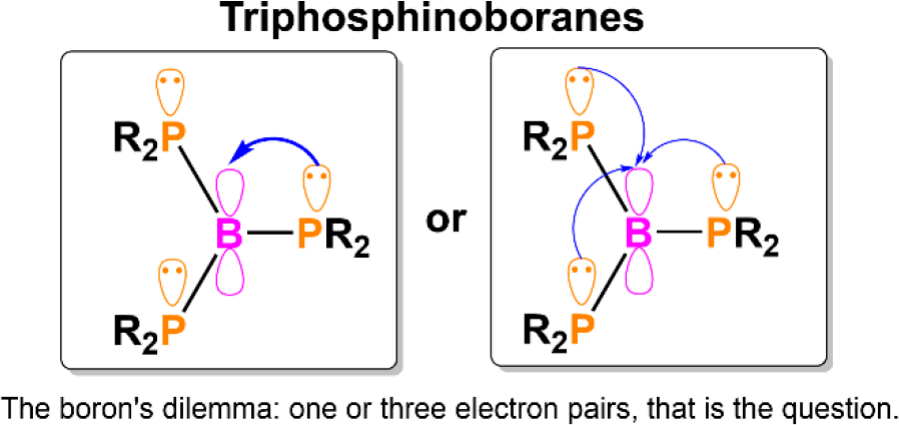

Herein, we present the synthesis of the first fully characterized monomeric triphosphinoboranes. The simple reaction of boron tribromide with 3 equiv of bulky lithium phosphide *t*Bu_2_PLi yielded triphosphinoborane (*t*Bu_2_P)_3_B. Triphosphinoboranes with diversified phosphanyl substituents were obtained via a two-step reaction, in which isolable bromodiphosphinoborane (*t*Bu_2_P)_2_BBr is first formed and then reacts with 1 equiv of less bulky phosphide R_2_PLi (R_2_P = Cy_2_P, *i*Pr_2_P, *t*BuPhP, or Ph_2_P). By utilizing this method, we obtained a series of triphosphinoboranes with the general formula (*t*Bu_2_P)_2_BPR_2_. On the basis of structural and theoretical studies, two main types of triphosphinoborane structures can be distinguished. In the first type, all three electron lone pairs interact with the formally empty p orbital of the central boron atom, resulting in delocalized π bonding, whereas in the second type, one localized P=B bond and two P–B bonds are observed. The Lewis acidic–basic properties of triphosphinoboranes during the reaction of (*t*Bu_2_P)_2_BP*i*Pr_2_ with H_3_B·SMe_2_ were analyzed. The P–B bond-containing compound mentioned above not only formed an adduct with BH_3_ but also activated the B–H bond of the borane molecule, resulting in the incorporation of the BH_2_ unit into two phosphorus atoms and migration of a hydride to the boron atom of the parent triphosphinoborane. The structures of the triphosphinoboranes were confirmed by single-crystal X-ray analysis, multinuclear nuclear magnetic resonance spectroscopy, and elemental analysis.

## Abstract 2

The synthesis and structural analysis of the first fully characterized monomeric triphosphinoboranes are presented.

## 1 Introduction

Nonmetallic systems containing directly linked phosphorus and boron atoms constitute a rapidly expanding area of research in modern chemistry, *inter alia*, due to their application in the activation of small molecules.^1−13^ These systems include tricoordinated boron and phosphorus compounds, namely, phosphinoboranes and diphosphinoboranes.^14,15^

**Chart 1:**
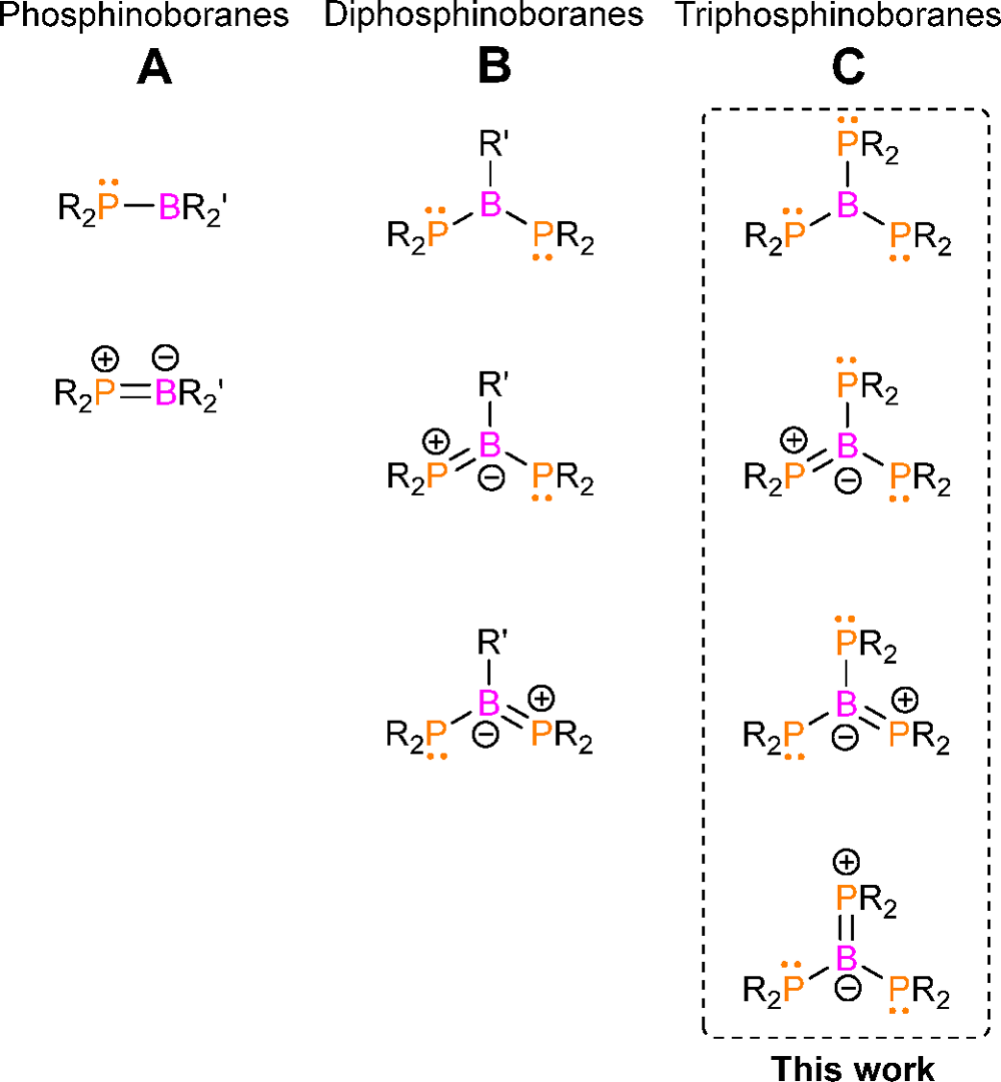
Possible Lewis Structures of Compounds with P–B Bonds

The geometry of P–B systems and their electronic structure are vital for their reactivity. Phosphinoboranes can be divided into two groups. In the first group, the phosphorus and boron atoms are planar and the distance between these atoms is relatively short (double-bond character); in the second group, the P–B bond has a single-bond character, and the phosphorus atom is pyramidal. Most phosphinoboranes have structures somewhere between these two extremes (Chart 1A).^16^

Interestingly, these species can be viewed as intramolecular frustrated Lewis pairs. Stephan showed that phosphinoboranes of the first type, such as R_2_PB(C_6_F_5_)_2_ (R = *t*Bu or Cy), can be used in the activation of H_2_ and the dehydrogenation of ammonia borane.^12^ However, these systems do not activate CO_2_, and we showed that this is in line with the electronic structure of the P–B system used, where the lone pair on the P atom is not available for an electrophilic reagent.^17^ Moreover, we designed and synthesized diaminophosphinoboranes possessing a single P–B bond and an accessible lone pair on the P atom that are capable of activating CO_2_,^17^ N_2_O, and SO_2_^18^ under very mild conditions. Recently, the Westcott group reported the synthesis of additional P–B systems named phosphinoboronate esters R_2_PBpin (R = Ph or Cy; Bpin = pinacolborane)^2^ and Ph_2_PBcat (Bcat = catecholborane); they also reported a phosphinoboration reaction in collaboration with the Stephan group. Broad applicability was shown by applying 1,2-additions to a variety of unsaturated organic compounds: aldehydes, ketones, imines,^2,3^ N-heterocycles,^4^ heteroallenes,^5^ diazobenzene,^6^ diazomethanes,^7,8^ acyl chlorides,^9^ and alkynes.^10^ These researchers also showed the application of R_2_PBpin, R_2_PBMes_2_, and R_2_PBcat [R = *t*Bu, Ph, or Mes (Mes = 1,3,5-Me_3_C_6_H_2_)] in reactions with CO_2_, resulting in the formation of R_2_PCO_2_BR′_2_ species. Additionally, access to diphospha-ureas was provided by Bcat-containing B/P reagents resulting from double 1,2-phospha-addition to CO_2_.^5,11^

The chemistry of diphosphinoboranes has been explored to a lesser extent than that of other P–B systems (Chart 1B). Most synthesis attempts have been made by Nöth et al.^19−21^ Recently, we vastly expanded the chemistry of these compounds, not only by the synthesis and isolation of several new diphosphinoboranes^22^ but also by revealing their potential in the activation of small molecules.^23^ Our preliminary research on the reactivity of selected diphosphinoboranes revealed that these species react with isocyanates, CO_2_, and H_2_. We have also reported the very first P–B system that activates both H_2_ and CO_2_.^23^

Having described the straightforward synthetic route and application of diaminophosphinoboranes and diphosphinoboranes, naturally, we decided to investigate the potential of triphosphinoboranes (Chart 1C). To date, there have been no full reports on the synthesis and isolation of species with the general formula (R_2_P)_3_B. In the literature, only a single report on the synthesis of triphosphinoborane (Mes_2_P)_2_BPMe_2_ is available, and the structure was confirmed only via ^31^P and ^11^B NMR spectroscopy.^24^ This compound was obtained in the reaction of (Mes_2_P)_2_BBr with Me_2_PLi in toluene. The presence of (Mes_2_P)_3_B after the reaction of BBr_3_ with Mes_2_PLi, along with other products, was also reported and confirmed by ^31^P NMR.

Herein, we present a series of the first fully characterized triphosphinoboranes with diversified substituents on the P atoms. Moreover, we elucidate the influence of steric hindrance and the electronic character of the phosphanyl groups on the structure of triphosphinoboranes.

## 2 Results and Discussion

**Scheme 1:**
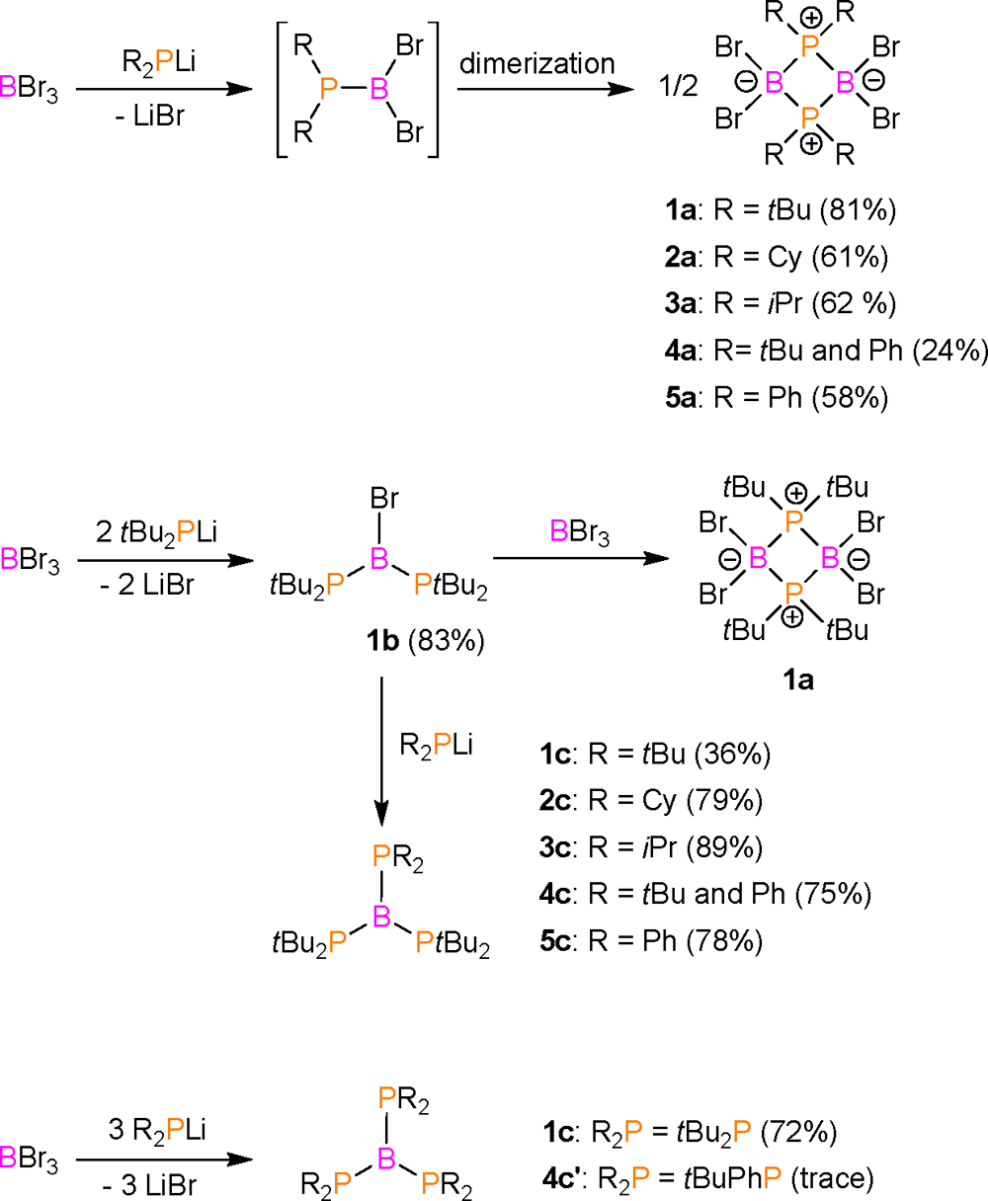
Syntheses of Phosphinoborane Dimers (**1a–5a**), Diphosphinoborane (**1b**), and Triphosphinoboranes (**1c–5c**)

To synthesize triphosphinoboranes with the same phosphanyl substituents, we chose a simple method involving the reaction of boron tribromide with lithium phosphides. As precursors of the phosphorus fragments, we used lithium phosphides with diversified substituents on the P atoms such as *t*Bu_2_PLi (**1**), Cy_2_PLi (**2**), *i*Pr_2_PLi (**3**), *t*BuPhPLi (**4**), and Ph_2_PLi (**5**).^25^ Thus, we studied the stoichiometry of the reactions of lithium phosphides with boron tribromide. The reactions of equimolar amounts of these reagents in toluene afforded cyclic dimers of 1,1-dibromophosphinoboranes (**1a–5a**) (Scheme 1). The NMR signatures of these species were very similar to those of previously reported 1,1-dihalogenophosphinoborane dimers (Table 1).^1,26^**1a** was previously synthesized in the reaction of *t*Bu_2_PH·BBr_3_ with LiN(SiMe_3_)_2_, and its structure was fully confirmed by NMR spectroscopy and X-ray crystallography.^26^ Bullen and co-workers reported the synthesis of **5a** and its iodo derivative by the reaction of Ph_2_PH with BX_3_ (X = Br or I) in the presence of Et_3_N; however, spectroscopic data for **5a** were not provided.^27,28^ Synthetic access to dichlorophosphinoborane dimers was described by Stephan and co-workers, where R_2_P=B(C_6_F_5_)_2_ (R = *t*Bu or Cy) reacted with BCl_3_ to form (R_2_P-BCl_2_)_2_ and ClB(C_6_F_5_)_2_.^1^ Analytically pure samples of **2a–5a** were isolated at −30 °C from a concentrated toluene solution as colorless crystals. The structures of **2a–5a** were confirmed by X-ray diffraction (Figures S1–S4).

**Table 1:** ^31^P{^1^H} and ^11^B NMR Data of Phosphinoborane Dimers **1a–5a**

compound	δ_P_ (ppm)	δ_B_ (ppm)	^1^*J*_PB_ (Hz)
**1a**(26)	7.0 (sept, *t*Bu_2_P)	–8.2 (t)	86
**2a**	–24.4 (sept, Cy_2_P)	–10.7 (t)	96
**3a**	–15.8 (sept, *i*Pr_2_P)	–10.8 (t)	96
**4a**	–11.1 (sept, *t*BuPhP)	–10.2 (t)	92
**5a**	–24.2 (sept, Ph_2_P)	–10.0 (t)	97

**Figure 1:**
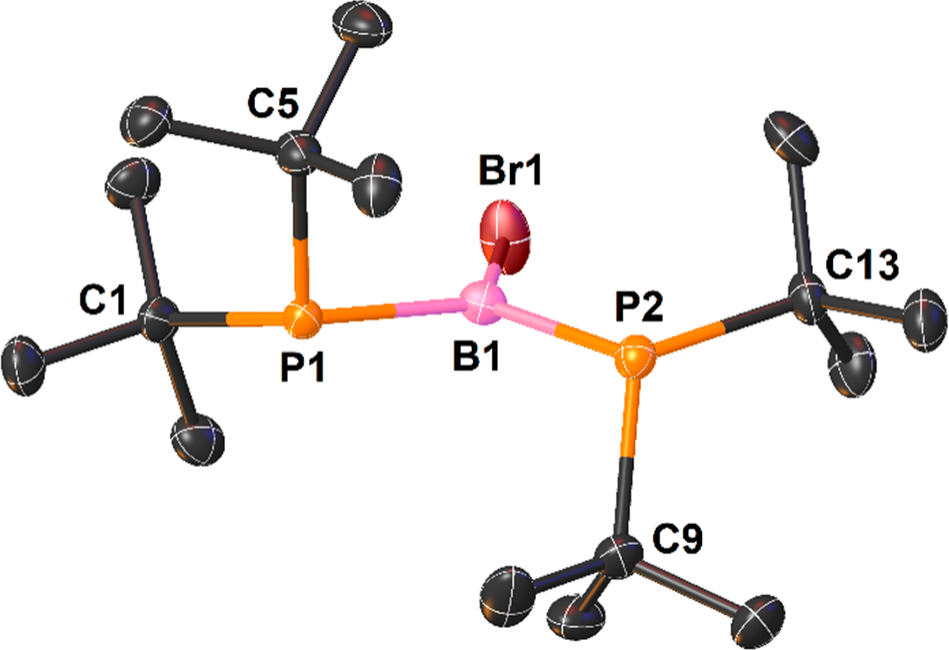
View of the molecular structure of **1b** (50% probability ellipsoids, H atoms omitted).

Next, we tested the reactivity of boron tribromide with an excess of lithium phosphides. The addition of a toluene solution of BBr_3_ to 2 equiv of *t*Bu_2_PLi (**1**) suspended in toluene at −50 °C led to the immediate formation of bromodiphosphinoborane **1b** and a LiBr precipitate, together with small amounts of *t*Bu_2_PH and (*t*Bu_2_P)_2_ (products of hydrolysis and radical side reactions). The ^11^B NMR spectra of **1b** displayed a downfield-shifted broad singlet at 74.6 ppm, indicating a tricoordinated boron center (Table 2). The ^31^P{^1^H} NMR spectrum of **1b** consists of only one broad singlet at 46.2 ppm, which confirms the equivalence of both phosphorus atoms. Compound **1b** crystallized from a concentrated petroleum ether solution at −30 °C as red crystals in 83% yield. The formation of monomeric **1b** was confirmed by single-crystal X-ray diffraction (Figure 1). The central B1 atom is connected to the P1 and P2 atoms of the *t*Bu_2_P phosphanyl groups and the Br1 atom. The geometry around the B1 atom is planar (sum of the angles around the B atom ∑B1 = 360 °C), whereas both phosphanyl groups exhibit pyramidal geometry, with a sum of angles around the P atoms of approximately 324°. The phosphorus–boron distances are approximately 1.91 Å, which are shorter than the expected lengths for single, covalent P–B bonds [sum of the single bond covalent radii for P and B ∑*r*_cov_(P–B) = 1.96 Å].^29^

**Table 2:** ^31^P{^1^H} and ^11^B NMR Data of Diphosphinoborane (**1b**) and Triphosphinoboranes (**1c–5c**)

compound	δ_P_ (ppm)	^2^*J*_PP_ (Hz)	δ_B_ (ppm)
**1b**	46.2 (bs, *t*Bu_2_P)	–	74.6 (bs)
**1c**	40.8 (bs, *t*Bu_2_P)	–	56.3 (bs)
**2c**	122.4 (bs, Cy_2_P); −1.5 (bs, *t*Bu_2_P)	–	50.4 (bs)
**3c**	130.3 (bs, *i*Pr_2_P); −2.9 (bs, *t*Bu_2_P)	–	50.6 (bs)
**4c**	85.5 (bs, *t*BuPhPB); 12.4 (bd, *t*Bu_2_P)	106	59.6 (bs)
**5c**	52.3 (bd, *t*Bu_2_P); −12.4 (bt, Ph_2_P)	98	63.8 (bs)

In contrast to the reaction involving *t*Bu_2_PLi, the reactions of BBr_3_ with a 2-fold excess of less bulky lithium phosphides (**2–5**) exclusively afforded dimers **2a–5a**, respectively. Notably, in the case of the reaction using **1**, the reverse addition of substrates afforded only analogous dimer **1a**. Moreover, the equimolar reaction of **1b** with BBr_3_ also yielded dimer **1a** (Scheme 1).

Next, we studied the influence of a 3-fold excess of phosphorus reagents on the outcome of reactions with BBr_3_. In experiments involving *t*Bu_2_PLi, NMR spectroscopy revealed the formation of **1b** together with new compound **1c**, exhibiting broad ^11^B NMR and ^31^P{^1^H} resonances at 56.3 and 40.8 ppm, respectively (Scheme 1 and Table 2).

**Figure 2:**
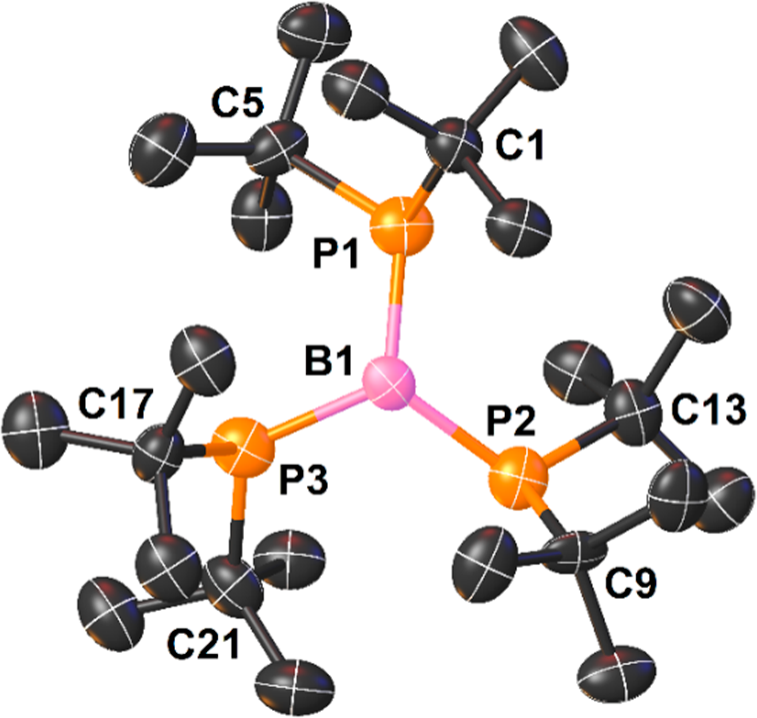
View of the molecular structure of **1c** (50% probability ellipsoids, H atoms omitted). One molecule from 4/3 molecules present in the independent part of the unit cell is shown.

After 24 h, the signals of **1b** disappeared, and only resonances attributed to **1c** were present in the ^11^B and ^31^P{^1^H} NMR spectra of the reaction mixture. This observation suggested that **1b** is the intermediate compound that further reacts with the third equivalent of *t*Bu_2_PLi to form triphosphinoborane **1c**. The downfield-shifted resonance in the ^11^B spectra of **1c** is consistent with the trigonal planar environment of the boron atom, whereas the presence of only one resonance in the ^31^P{^1^H} spectra of **1c** agrees with the structure with three equivalent *t*Bu_2_P groups bound to one boron atom. **1c** was isolated by low-temperature crystallization from petroleum ether as red crystals in 72% yield. X-ray structure analysis confirmed the formation of triphosphinoborane **1c** (Figure 2).

The analogous reactions involving a 3-fold excess of less bulky phosphides **2–5** afforded dimers **2a–5a**, respectively, as the main products. Interestingly, during the reaction using *t*BuPhPLi (**4**), in addition to main product **4a**, trace amounts of triphosphinoborane **4c′** were formed (Scheme 1). A few crystals of **4c′** were grown at low temperature from the petroleum ether solution obtained from washing the crude product (**4a** is only slightly soluble in petroleum ether). We assumed that the concentration of **4c′** in the reaction mixture must be very low, as this product could not be detected using our NMR spectrometer. Moreover, our attempts to optimize the synthesis to obtain significant amounts of **4c′** were unsuccessful. Therefore, **4c′** was characterized using only single-crystal X-ray diffraction (Scheme S5).

The successful isolation of bromodiphosphinoborane **1b** encouraged us to use this compound in the synthesis of triphosphinoboranes. To our delight, **1b** reacted with phosphides **2–5** with the clean formation of new triphosphinoboranes **2c–5c**, respectively, as the main products (Scheme 1). The reactions mentioned above proceeded in toluene and even faster in diethyl ether, where the complete conversion of parent **1b** into triphosphinoboranes was observed within 24 h. The ^11^B NMR spectra of **2c–5c** consist of a broad resonance in the range of 50.4–63.8 ppm, confirming the formation of monomeric species and the presence of tricoordinated boron atoms (Table 2). The ^31^P{^1^H} spectra of **2c–5c** show two resonances, one attributed to two equivalent P1 and P2 atoms of *t*Bu_2_P groups and the other assigned to the P3 atom of a less bulky phosphanyl group such as Cy_2_P (**2c**), *i*Pr_2_P (**3c**), *t*BuPhP (**4c**), or Ph_2_P (**5c**). In the case of **2c–4c**, the ^31^P{^1^H} resonance of the *t*Bu_2_P group (P1 and P2) has a value ranging from −2.9 to 12.4 ppm, and the P3 resonance of the less bulky phosphanyl group is shifted strongly downfield, with values from 85.5 to 130.3 ppm (Table 2). Interestingly, such a strong downfield shift of phosphorus resonances is not observed for triphosphinoborane **5c**. Moreover, in comparison to those of **2c–4c**, the relative position of the signals in the ^31^P{^1^H} spectrum of **5c** is inverted, where the most downfield signal is attributed to the P1 and P2 atoms of the *t*Bu_2_P group (52.3 ppm), whereas the P3 atom of the Ph_2_P group resonates at a higher field (−12.4 ppm). Notably, in the ^31^P{^1^H} spectra of **4c** and **5c**, ^2^*J*_PP_ coupling is observed, with values of 106 and 98 Hz, respectively. In the case of other triphosphinoboranes, such coupling was not observed because of the broadness of the signals (**2c** and **3c**) or the equivalence of the three phosphorus atoms (**1c**).

According to the studies of Power and co-workers, ^31^P NMR spectroscopy is a very useful tool for the analysis of π interactions in tricoordinated compounds possessing direct P–B bonds.^30^ They showed that the large positive value of the chemical shift indicates a significant π interaction between the P and B atoms. Therefore, the strongly downfield-shifted resonances of P3 in the ^31^P{^1^H} spectra of **2c–4c** suggest the presence of localized multiple bonds between the P3 atom and the B1 atom. Note that for **2c** and **3c**, the chemical shifts corresponding to the P3 atoms have values (122.4 and 130.3 ppm, respectively) even more positive than those observed for planar phosphinoboranes with P=B bonds possessing strongly electron-withdrawing groups at the B atom [δ = 120.7 ppm for *t*Bu_2_P=B(C_6_F_5_)_2_, and δ = 92.1 ppm for Cy_2_P=B(C_6_F_5_)_2_].^1^ On the contrary, in the case of **1c** and **5c**, delocalized π interactions between boron and three phosphorus atoms are expected on the basis of the ^31^P{^1^H} NMR data of these species. Compounds **2c–5c** were isolated in high yields as crystals from concentrated petroleum ether solutions at −30 °C. Due to the presence of reactive P–B bonds and the low coordination number of both phosphorus and boron centers, triphosphinoboranes **1c–5c** rapidly decompose when in contact with air.

The molecular structures of all obtained triphosphinoboranes (**1c–5c**) were determined by single-crystal X-ray diffraction. The most important parameters of **1c–5c** are listed in Table 3. Moreover, to further investigate the electronic structure of triphosphinoboranes, NBO analysis of these species was performed. Depending on the geometry and electronic structure, two main types of triphosphinoboranes can be distinguished. The compounds within the mentioned groups exhibit common structural features; therefore, they will be discussed together. The first group includes triphosphinoboranes **1c** and **4c′**, and their molecular structures are presented in Figure 2 and Figure S5, respectively. They exhibit an almost planar geometry around the B1 atom (for **1c**, ∑B1 = 353.3°; for **4c′**, ∑B1 = 352.3°), whereas the geometry around all phosphorus atoms is similar and is intermediate between ideal planar and pyramidal (for **1c**, average ∑P = 342.8°; for **4c′**, average ∑P = 329.9°). All three P–B bonds have comparable lengths slightly longer than 1.90 Å, which are between the expected distances for single and double phosphorus–boron bonds [∑*r*_cov_(P–B) = 1.96 Å, and ∑*r*_cov_(P=B) = 1.80 Å].^29,31^

**Table 3:** Selected Bond Lengths and Geometries around the B1 and P1–P3 Atoms for Triphosphinoboranes **1c–5c**^a^

compound	B1–P1 (Å) [WBI]	B1–P2 (Å) [WBI]	B1–P3 (Å) [WBI]	∑B1 (deg)	∑P1, ∑P2, ∑P3 (deg)
**1c**	1.904(7)^b^ [1.22]	1.936(9)^b^ [1.22]	1.913(7)^b^ [1.22]	353.3^b^	343.4,^b^ 342.3,^b^ 342.7^b^
**2c**	2.010(5) [1.00]	2.021(3) [1.00]	1.799(2) [1.70]	359.8	327.6, 324.3, 359.9
**3c**	1.990(2) [1.00]	1.990(1) [1.00]	1.792(1) [1.70]	360.0	328.2, 322.8, 359.6
**4c**	1.990(1) [1.02]	1.989(1) [1.00]	1.810(1) [1.66]	359.8	326.9, 323.8, 358.9
**4c′**	1.918(2) [1.20]	1.904(2) [1.23]	1.911(2) [1.22]	352.3	329.1, 330.2, 330.5
**5c**	1.953(2) [1.10]	1.881(2) [1.35]	1.899(2) [1.22]	354.5	329.9, 345.3, 336.6

a The Wiberg bond indices (WBIs) for the corresponding bonds are provided in brackets.

b Average values for 4/3 molecules present in the independent part of the unit cell.

**Figure 3:**
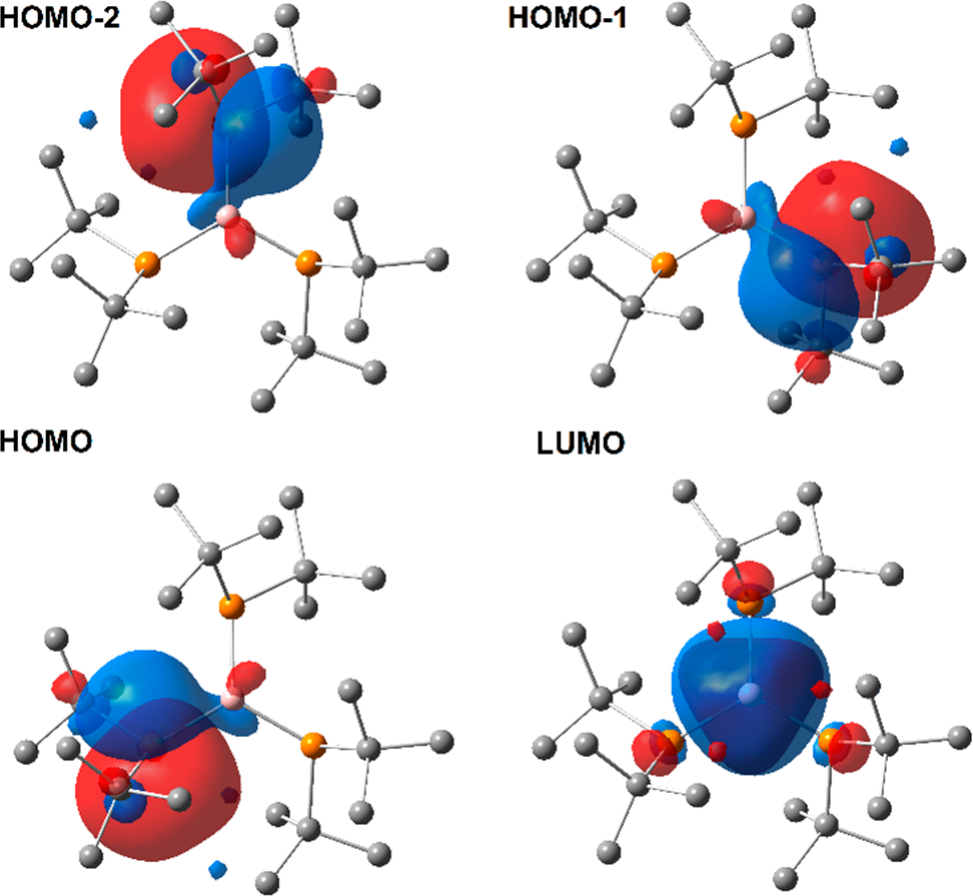
View of NBOs of **1c** engaged in donor–acceptor π interactions.

For **1c** and **4c′**, the relatively short P–B distances and the high degree of planarity of R_2_P moieties suggested the interaction of a formally empty p orbital on boron with three electron pairs on the P1–P3 atoms. Indeed, NBO analysis confirmed this assumption. The calculated P–B bond orders for **1c** and **4c′** are equal to or slightly greater than 1.20, indicating the partial multiple characters of these bonds. The second-order perturbation analysis provides additional information about the π interactions between the Lewis acidic B center and the three Lewis basic P centers (Table 4). The stabilizing energies *E*(2), which characterize donor–acceptor interactions between the B and P centers, have values of approximately 15 kcal/mol for **1c** and between 17.83 and 20.93 kcal/mol for **4c′**. Moreover, the triphosphinoboranes of this group exhibit decreased occupancies of the orbitals associated with electron pairs on the P atoms and increased occupancies of the unhybridized p orbitals of the central B atoms. The NBO orbitals of **1c** involved in P–B π interactions are presented in Figure 3. The NLMO analysis of **1c** and **4c′** further corroborates the presence of π donation from all three P atoms to the formally empty p orbital of boron and reveals the significant contribution of the B atom (7–13%) in NMLOs attributed to electron lone pairs at P atoms. All of these observations confirm the almost equal and significant π contributions in all three P–B bonds in the triphosphinoboranes in the first group.

**Table 4:** Stabilizing Energies *E*(2) Associated with Electron Delocalization between Donor P Centers and Acceptor B Centers in **1c–5c**

compound	donor	occupancy	acceptor	occupancy	*E*(2) (kcal/mol)
**1c**	LP(P1)	1.71	LP*(B1)	0.65	14.97
	LP(P2)	1.71			15.14
	LP(P3)	1.71			15.04
**2c**	LP(P1)	1.89	σ*(P3–B1)	0.05	8.66
	LP(P2)	1.90	σ*(P1–B1)	0.05	7.65
**3c**	LP(P1)	1.89	σ*(P3–B1)	0.05	8.98
	LP(P2)	1.90	σ*(P1–B1)	0.05	8.32
**4c**	LP(P1)	1.89	σ*(P3–B1)	0.06	9.92
	LP(P2)	1.90	σ*(P1–B1)	0.05	7.87
**4c′**	LP(P1)	1.76	LP*(B1)	0.61	17.83
	LP(P2)	1.74			20.93
	LP(P3)	1.74			19.18
**5c**	LP(P1)	1.84	LP*(B1)	0.64	3.06
	LP(P2)	1.64			29.33
	LP(P3)	1.66			28.16

**Figure 4:**
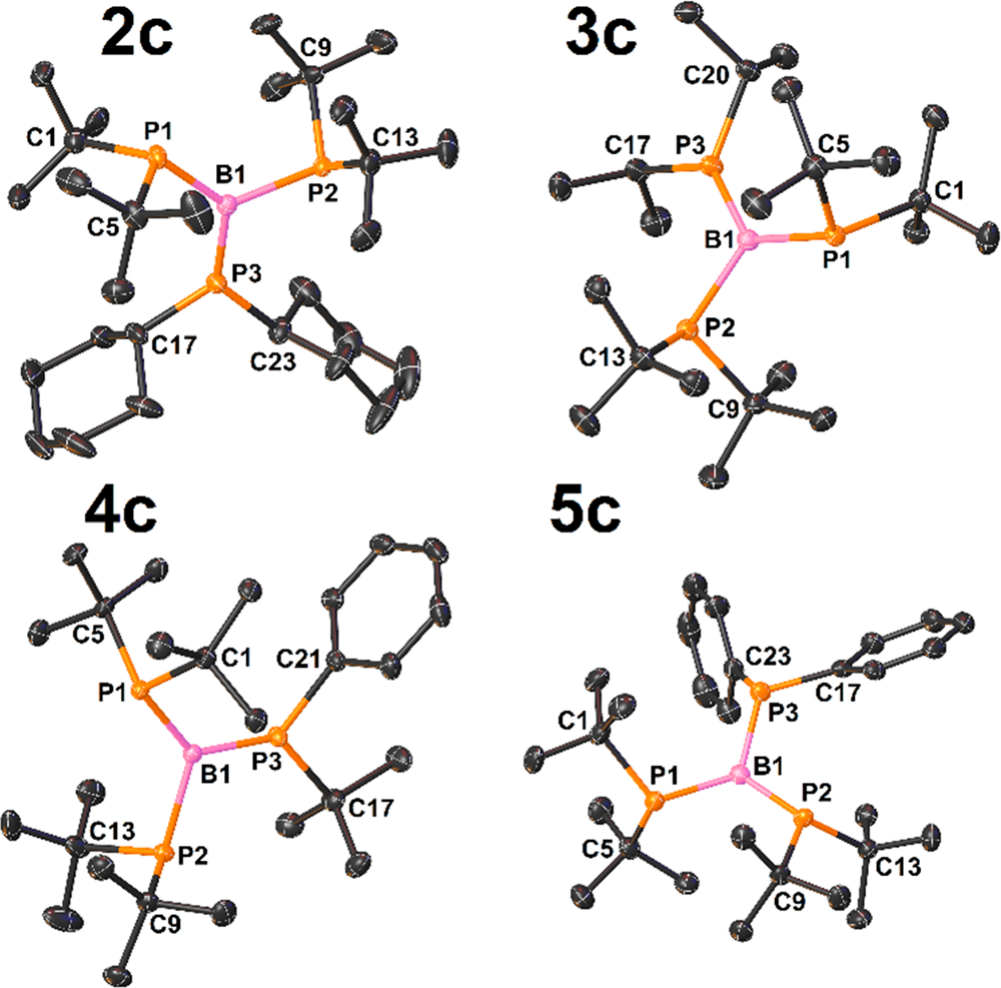
View of the molecular structures of **2c–5c** (50% probability ellipsoids, H atoms omitted).

**Figure 5:**
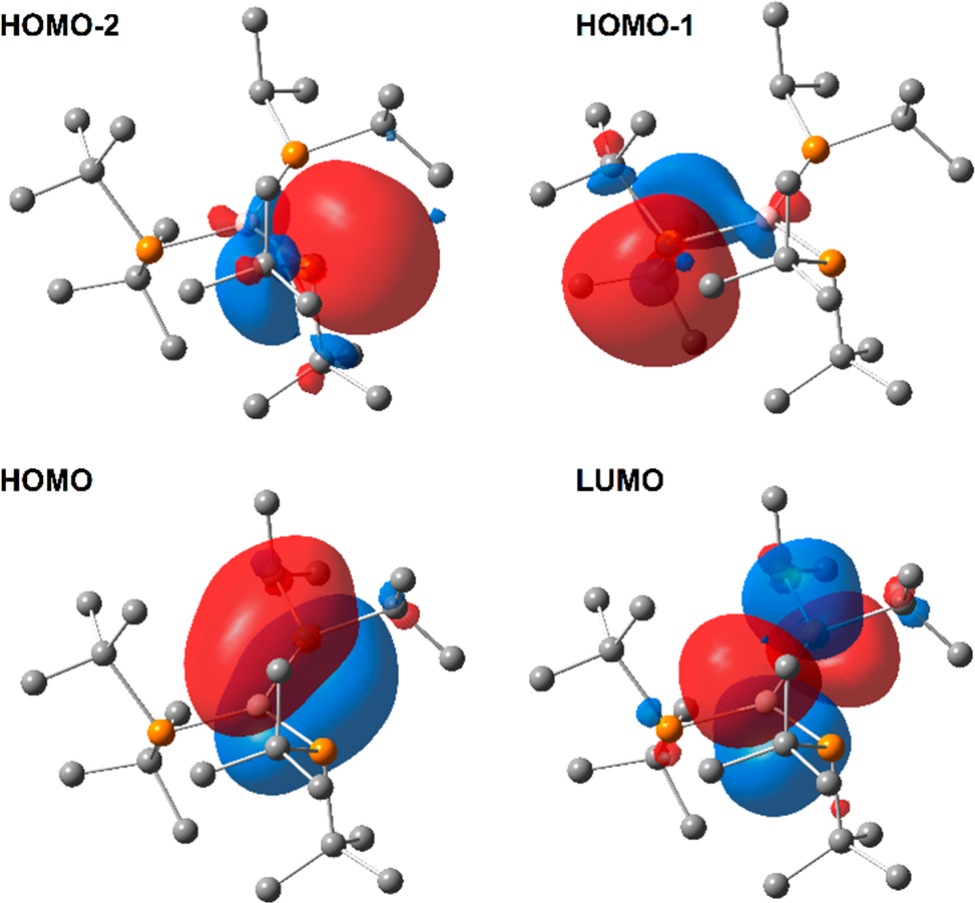
View of the NBOs of **3c** associated with π(P3–B1) and π*(P3–B1) orbitals, and electron lone pairs at the P1 and P2 atoms.

Compounds **2c–4c** represent the second group of triphosphinoboranes. Their molecular structures are presented in Figure 4. The most striking features of the triphosphinoboranes in this group are the planar geometry around the B1 and P3 atoms and the pyramidal geometry around the P1 and P2 atoms (Table 3). Furthermore, in contrast to those of the triphosphinoboranes in the first group, the P–B bond lengths in the second group are more diversified, with very long P1–B1 and P2–B1 distances of approximately 2.00 Å, a distance even slightly longer than the length of a typical single covalent bond [∑*r*_cov_(P=B) = 1.96 Å],^29^ and very short [1.792(1)–1.810(1) Å] P3–B1 distances indicative of the double-bond character of these bonds [∑*r*_cov_(P=B) = 1.80 Å].^31^ The NBO analysis of **2c–4c** confirmed the presence of localized π bonds between the P3 and B1 atoms (Figure 5). As expected, the NBO π(P3–B1) orbitals of **2c–4c** dominate the contribution of the P3 atom (68–70%). Moreover, the calculated Wiberg bond orders for P3–B1 bonds have large values ranging from 1.66 to 1.70, whereas the obtained bond orders for the P1–B1 and P2–B1 bonds are very close to 1 (Table 3). These findings corroborate the ^31^P{^1^H} NMR spectroscopic data of **2c–4c**, where strongly downfield-shifted resonances of P3 atoms were observed.

Moreover, weak interactions were found between the electron lone pairs on the P1 and P2 atoms and the antibonding σ*(P3–B1) and σ*(P1–B1) orbitals for the structures of the second type (Table 4). In the case of **2c–4c**, the lack of π donation from P1 and P2 atoms is additionally confirmed by the very small contribution of the boron atom (1%) in NMLOs associated with electron lone pairs of the mentioned phosphorus atoms. The influence of the steric effect of substituents on the P atoms on the structures of the triphosphinoboranes is clear. The introduction of substituents smaller than *t*Bu groups at the P3 atom allows sp^2^ hybridization of the P3 atom and formation of localized P3=B1 bonds.

Interestingly, **5c** combines the structural features of triphosphinoboranes of both aforementioned groups. The X-ray structure of **5c** is depicted in Figure 4. Similar to the compounds in the first group, **5c** displays an almost planar geometry around the B1 atom and a high degree of planarity of all phosphorus atoms (Table 3). However, in contrast to that of the first group but similar to that of the second group, the geometry around the phosphorus atom and the phosphorus–boron distances are more diversified. In the case of **5c**, the planarity of the phosphanyl groups increases in the following order: *t*Bu_2_P1 < Ph_2_P3 < *t*Bu_2_P2. The phosphorus–boron distances decrease in the following order: P1–B3 > P3–B3 > P2–B3 (Table 3). NBO analysis of **5c** revealed that the electron pairs on P1–P3 interact with the Lewis acidic boron center to a different extent (Table 4). Calculations of the electron delocalization energies *E*(2) show that the strength of the interactions mentioned above increases in the following order: P1 → B3 (3.06 kcal/mol), P3 → B3 (28.16 kcal/mol), and P2 → B3 (29.33 kcal/mol). It is worth mentioning that the contribution of the B atom in NLMOs of **5c** attributed to electron lone pairs of phosphorus atoms is diversified [LP(P1), 3%; LP(P3), 10%; LP(P2), 17%] and indicates the strongest π donation from the P2 atom and the weakest from the P1 atom. Despite the less bulky phenyl groups at the P3 atom, the structure of **5c** does not contain a localized P3=B1 bond, similar to triphosphinoboranes of the second type. We assume that the electron-withdrawing properties of the phenyl groups weaken the donor abilities of the P3 atom; therefore, the extent of delocalization of the electron pair between the P3 and B1 atoms is smaller.

**Scheme 2:**
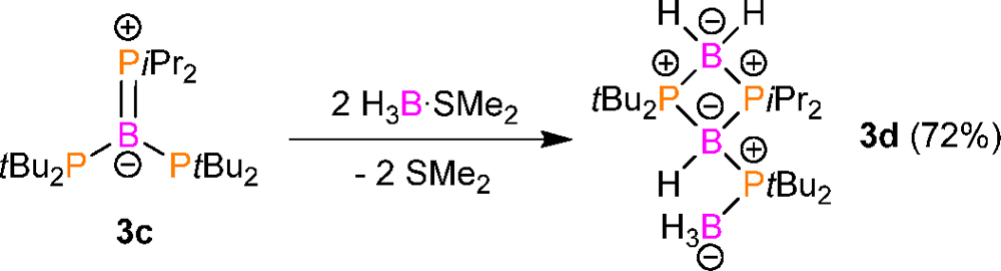
Reaction of **3c** with H_3_B·SMe_2_

The unusual structural features of triphosphinoboranes, such as the presence of three Lewis basic phosphorus atoms that are directly bound to the Lewis acidic boron center, encouraged us to test their reactivity toward simple adducts of Lewis bases and Lewis acids. For this study, we selected **3c**, which exhibits one double and two single P–B bonds and hence a diversified phosphanyl group geometry. **3c** reacted cleanly with 2 equiv of H_3_B·SMe_2_ in toluene to form **3d** (Scheme 2). The end point of the reaction was easily observed by the discoloration of the red toluene solution of **3c**. The reactions with a molar ratio of 1:1 afforded a mixture of **3c** and **3d**, whereas an experiment involving a large excess of borane adduct yielded **3d** and unreacted H_3_B·SMe_2_. NMR spectroscopic and X-ray diffraction studies indicated that **3c** not only forms a classical adduct with BH_3_ but also activates the B–H bonds within the BH_3_ moiety.

**Figure 6:**
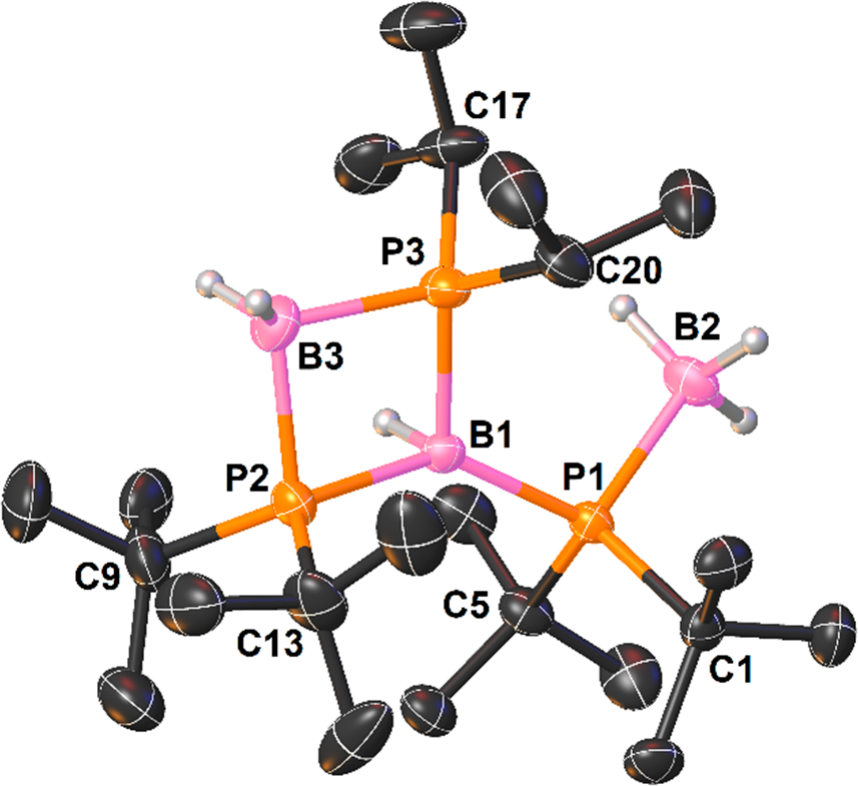
View of the molecular structure of **3d** (50% probability ellipsoids, H atoms, except those bonded to boron atoms, omitted).

The ^11^B NMR spectrum of **3d** displays a broad multiplet at −23.9 and two broad overlapping multiplets from −33.8 to −38.2 ppm, which are in the characteristic region for the tetracoordinated boron center. The presence of three very broad signals in the ^31^P{^1^H} NMR spectrum at 36.2, 29.5, and 4.7 ppm indicates three inequivalent P atoms in the structure of **3d**. In contrast to that of **3c**, the signal attributed to the phosphorus atom of the P*i*Pr_2_ group is shifted strongly upfield (**3c**, 130.3 ppm; **3d**, 4.7 ppm), suggesting a lack of P–B π interactions. Furthermore, the ^1^H NMR spectrum consists of very broad signals that can be attributed to hydrogen atoms directly bound to boron atoms (0.84–2.15 ppm, 5H; 3.37 ppm, 1H). X-ray-quality crystals of **3d** were grown from a petroleum ether solution at low temperatures. The molecular structure of **3d** is presented in Figure 6. An X-ray diffraction study revealed that the P1 atom formed a coordination bond with the B2 atom of the BH_3_ moiety. The second borane molecule reacted with **3c** to incorporate a BH_2_ unit between the P2 and P3 atoms and to cause the migration of a hydride moiety to the B1 atom. We assume that the B–H bond activation by **3c** proceeds via a stepwise mechanism, where the first step is the coordination of the BH_3_ molecule to a P atom of the *t*Bu_2_P group. Then hydride migrates to the B atom of the parent triphosphinoborane, followed by the coordination of the P atom of the *i*Pr_2_P group to the B atom of the BH_2_ unit. Notably, the hydrogen atoms of the BH, BH_2_, and BH_3_ units were located on the basis of analysis of the Fourier electron density map. It was previously confirmed by ^11^B NMR spectroscopy that all boron atoms are tetracoordinated. The same coordination number was observed for all P atoms. The P–B bond distances were found to be in the range expected for single covalent bonds [1.951(4)–2.025(3) Å], and no structural evidence for π interaction between P and B atoms was observed. B–H bond activation in BH_3_ compounds by nonmetallic systems is very rare. An example of this kind of reactivity is the 1,1-addition of BH_3_ to carbenoids^32^ or stable carbenes.^33^ To date, there have been no reports on BH_3_ activation by systems containing P–B bonds.

## 3 Conclusions

The obtained triphosphinoboranes exhibit unique structural features, where the Lewis acidic boron center is directly bonded to three Lewis basic phosphorus centers. We showed that the electronic and steric properties of phosphanyl substituents have a significant influence on the structure of triphosphinoboranes, where structures with delocalized or localized P–B π bonding were obtained. The ambiphilic nature of triphosphinoboranes together with the low coordination number of reactive phosphorus and boron centers makes these species very attractive in the activation of small molecules. The Lewis acidic–basic properties of triphosphinoboranes were manifested in the reaction with a borane adduct, where B–H bond activation was observed. Currently, reactivity investigations of these P–B bond systems toward a wide range of small inorganic and organic molecules are in progress.

## 4 Experimental Section

### General Information

All experiments were performed under an inert gas (argon) atmosphere. All manipulations were carried out using Schlenk, standard vacuum, and glovebox techniques. Petroleum ether, toluene, and diethyl ether were purified and dried using Na/K and benzophenone. C_6_D_6_ was purified with Na. Literature methods were followed for the synthesis of phosphides.^25^ BBr_3_ and BH_3_·SMe_2_ were purchased from commercial sources and used without further purification. A BBr_3_ solution in toluene and BMS (H_3_B·SMe_2_) in toluene were freshly prepared before use. NMR spectra were recorded on a Bruker Avance III HD 400 MHz spectrometer (external standards: TMS for ^1^H, and ^13^C, 85% aqueous H_3_PO_4_ for ^31^P, and BF_3_·Et_2_O for ^11^B) at ambient temperature.

Diffraction intensity data for all crystals were recorded on an IPDS 2T dual-beam diffractometer (STOE & Cie GmbH, Darmstadt, Germany) at 120.0(2) K with Mo Kα radiation from a microfocus X-ray source (GeniX 3D Mo High Flux, Xenocs, Sassenage, 50 kV, 1.0 mA, and λ = 0.71069 Å). The investigated crystals were thermostated under a nitrogen stream at 120 or 130 K using the CryoStream-800 device (Oxford CryoSystem) during the entire experiment.

Crystallographic data for all structures reported in this paper have been deposited with the Cambridge Crystallographic Data Centre as supplementary publications CCDC 2114349–2114360. The data can be obtained free of charge from The Cambridge Crystallographic Data Center via www.ccdc.cam.ac.uk/structures.

Elemental analyses were performed using a Vario El Cube CHNS apparatus at the University of Gdańsk. The lower value of carbon in elemental analyses of several compounds reported herein is caused by the extreme sensitivity of the triphosphinoboranes.

### Synthesis of (Cy_2_PBBr_2_)_2_ (**2a**)

To the suspension of 163 mg (0.8 mmol) of Cy_2_PLi in 4 mL of toluene was added dropwise 1 mL (0.8 mmol, 0.8 M) of a BBr_3_ solution in toluene at −30 °C. After the reaction mixture had been warmed to room temperature, toluene was evaporated under reduced pressure. The solid residue was partially dissolved in petroleum ether; LiBr was removed by filtration, and the solute was concentrated to a volume of 2 mL. The residue that was not soluble in petroleum ether was redissolved in toluene. Colorless crystals of **2a** (179 mg, 0.244 mmol, 61% yield) suitable for X-ray diffraction analysis were isolated from the concentrated toluene fraction at −30 °C: ^1^H NMR (C_6_D_6_, 400 MHz, 298 K) δ 1.06 (m, 4H, C**H**_2_), 1.28 (m, 8H, C**H**_2_), 1.59 (m, overlapped 20H, C**H**_2_), 2.58 (m, overlapped, 8H, C**H**_2_), 2.98 (m, 4H, PC**H**); ^11^B NMR (C_6_D_6_, 128 MHz, 298 K) δ −10.6 (t, ^1^*J*_PB_ = 96 Hz, Cy_2_P**B**Br_2_); ^31^P{^1^H} NMR (C_6_D_6_, 162 MHz, 298 K) δ −24.4 (sept, ^1^*J*_PB_ = 96 Hz, Cy_2_**P**BBr_2_), −28.0 (s, Cy_2_**P**H); ^13^C{^1^H} NMR (C_6_D_6_, 100 MHz, 298 K) δ 26.0 (s, **C**H_2_), 27.6 (t, *J*_CP_ = 5.6 Hz, **C**H_2_), 29.7 (t, *J*_CP_ = 2.5 Hz, **C**H_2_), 36.6 (t, ^1^*J*_CP_ = 13.8 Hz, P**C**H); elemental analysis calcd for C_24_H_44_B_2_Br_4_P_2_ (*M* = 735.79 g/mol) 39.18% C and 6.03% H, found 39.12% C and 6.07% H.

### Synthesis of (*i*Pr_2_PBBr_2_)_2_ (**3a**)

To the suspension of 100 mg (0.8 mmol) of *i*Pr_2_PLi in 4 mL of toluene was added dropwise the solution of 1 mL (0.8 mmol, 0.8 M) of BBr_3_ in toluene at −30 °C. After the reaction mixture had been warmed to room temperature, toluene was evaporated under reduced pressure. The solid residue was partially dissolved in petroleum ether; LiBr was removed by filtration, and the solute was concentrated to a volume of 2 mL. The residue that was not soluble in petroleum ether was redissolved in toluene. Colorless crystals of **3a** (175 mg, 0.246 mmol, 62% yield) suitable for X-ray diffraction analysis were isolated from the concentrated toluene fraction at −30 °C: ^1^H NMR (C_6_D_6_, 400 MHz, 298 K) δ 1.29 (dd, ^3^*J*_HH_ = 7 Hz, ^3^*J*_PH_ = 15 Hz, 24H, (C**H**_3_)_2_CH), 2.96 (m, 4H, (CH_3_)_2_C**H**); ^11^B NMR (C_6_D_6_, 128 MHz, 298 K) δ −10.7 (t, ^1^*J*_PB_ = 96 Hz, *i*Pr_2_P**B**Br_2_); ^31^P{^1^H} NMR (C_6_D_6_, 162 MHz, 298 K) δ −15.8 (sept, ^1^*J*_PB_ = 96 Hz, (*i*Pr_2_**P**BBr_2_)); ^13^C{^1^H} NMR (C_6_D_6_, 100 MHz, 298 K) δ 19.3 (broad s, (**C**H_3_)_2_CH), 25.6 (t, ^1^*J*_CP_ = 14 Hz, (CH_3_)_2_**C**H); elemental analysis calcd for C_12_H_28_B_2_Br_4_P_2_ (*M* = 575.54 g/mol) 25.04% C and 4.90% H, found 25.32% C and 4.61% H.

### Synthesis of (*t*BuPhPBBr_2_)_2_ (**4a**)

To the suspension of 136 mg (0.8 mmol) of *t*BuPhPLi in 4 mL of toluene was added dropwise the solution of 1 mL (0.8 mmol, 0.8 M) of BBr_3_ in toluene at −30 °C. After the reaction mixture had been warmed to room temperature, toluene was evaporated under reduced pressure. The solid residue was partially dissolved in petroleum ether; LiBr was removed by filtration, and the solute was concentrated to a volume of 2 mL. The residue that was not soluble in petroleum ether was redissolved in toluene. Colorless crystals of **4a** (64 mg, 0.096 mmol, 24% yield; lower crystallization yield due to the partial solubility of **4a** in petroleum ether) suitable for X-ray diffraction analysis were isolated from the concentrated toluene fraction at −30 °C: ^1^H NMR (C_6_D_6_, 400 MHz) δ 1.41 (m, 18H, (C**H**_3_)_3_CPPh), 6.94 (m, overlapped, 6H, C-**H**_**para**_ and C-**H**_**meta**_), 7.90 (m, 4H, C-**H**_**ortho**_); ^11^B NMR (C_6_D_6_, 128 MHz, 298 K) δ −10.2 (t, ^1^*J*_PB_ = 92 Hz, (*t*BuPhP**B**Br_2_)_2_); ^31^P{^1^H} NMR (C_6_D_6_, 162 MHz, 298 K) δ −5.7 (s, *t*BuPh**P**H, 2.7%), −11.1 (sept, ^1^*J*_PB_ = 92 Hz, *t*BuPh**P**BBr_2_); ^13^C{^1^H} NMR (C_6_D_6_, 100 MHz, 298 K) δ 28.2 (broad s, (**C**H_3_)_3_C), 38.6 (d, overlapped, ^2^*J*_CP_ = 11 Hz, (**C**H_3_)_3_C), 38.7 (d, overlapped, ^2^*J*_CP_ = 11 Hz, (**C**H_3_)_3_C), 124.6 (t, ^1^*J*_CP_ = 33 Hz, **C**_**i**_), 126.5 (t, ^2^*J*_CP_ = 5 Hz, **C**_**o**_), 130.5 (broad s, **C**_**p**_), 138.3 (broad s, **C**_**m**_); elemental analysis calcd for C_20_H_28_B_2_Br_4_P_2_ (*M* = 671.62 g/mol) 35.78% C and 4.20% H, found 35.62% C and 4.46% H.

### Synthesis of (Ph_2_PBBr_2_)_2_ (**5a**)

To the suspension of 96 mg (0.5 mmol) of Ph_2_PLi in 4 mL of toluene was added dropwise the solution of 0.625 mL (0.5 mmol, 0.8 M) of BBr_3_ in toluene at −30 °C. After the reaction mixture had been warmed to room temperature, toluene was evaporated under reduced pressure. The solid residue was partially dissolved in petroleum ether; LiBr was removed by filtration, and the solute was concentrated to a volume of 2 mL. The residue that was not soluble in petroleum ether was redissolved in toluene. Colorless crystals of **5a** (165 mg, 0.232 mmol, 58% yield) suitable for X-ray diffraction analysis were isolated from the concentrated toluene fraction at −30 °C: ^1^H NMR (C_6_D_6_, 400 MHz, 298 K) δ 6.92 (m, overlapped, 12H, C-**H**_**para**_ and C-**H**_**meta**_), 7.96 (m, 8H, C-**H**_**ortho**_); ^11^B NMR (C_6_D_6_, 128 MHz, 298 K) δ −10.0 (t, ^1^*J*_PB_ = 97 Hz, Ph_2_P**B**Br_2_); ^31^P{^1^H} NMR (C_6_D_6_, 162 MHz, 298 K) δ −24.2 (sept, ^1^*J*_PB_ = 97 Hz, Ph_2_**P**BBr_2_), −40.7 (s, Ph_2_**P**H); ^13^C{^1^H} NMR (C_6_D_6_, 100 MHz, 298 K) δ 126.4 (t, ^1^*J*_CP_ = 30 Hz, **C**_**i**_), 128.5 (t, ^2^*J*_CP_ = 6 Hz, **C**_**o**_), 131.2 (s, **C**_**p**_), 134.8 (t, ^3^*J*_CP_ = 4 Hz, **C**_**m**_); elemental analysis calcd for C_24_H_20_B_2_Br_4_P_2_ (*M* = 711.60 g/mol) 40.51% C and 2.83% H, found 40.61% C and 2.98% H.

### Synthesis of (*t*Bu_2_P)_2_BBr (**1b**)

To the suspension of 608 mg (4.0 mmol) of *t*Bu_2_PLi in 6 mL of toluene was added the solution of 2.5 mL (2.0 mmol, 0.8 M) of BBr_3_ in toluene at −50 °C. After the reaction mixture had been warmed to room temperature, toluene was evaporated under reduced pressure. The solid residue was dissolved in petroleum ether; LiBr was removed by filtration, and the solute was concentrated to a volume of 2 mL. Red crystals of **1b** (0.641 g, 1.67 mmol, 83% yield) were isolated at −30 °C: ^1^H NMR (C_6_D_6_, 400 MHz, 298 K) δ 1.50 (broad m, overlapped, 36H, (C**H**_3_)_3_C); ^11^B NMR (C_6_D_6_, 128 MHz, 298 K) δ 74.7 (broad s, (*t*Bu_2_P)_2_**B**Br); ^31^P{^1^H} NMR (C_6_D_6_, 162 MHz, 298 K) δ 46.3 (broad s, *t*Bu_2_**P**B), 39.7 (s, (*t*Bu_2_**P**)_2_, 5.4%), 19.5 (s, *t*Bu_2_**P**H, 1.3%); ^13^C{^1^H} NMR (C_6_D_6_, 100 MHz, 298 K) δ 33.2 (d, overlapped, ^3^*J*_CP_ = 6 Hz, (**C**H_3_)_3_C), 33.3 (d, overlapped, ^2^*J*_CP_ = 6 Hz, (**C**H_3_)_3_C), 36.7 (dd, ^1^*J*_CP_ = 6 Hz, ^2^*J*_CP_ = 2 Hz, (CH_3_)_3_**C**); elemental analysis calcd for C_16_H_36_BBrP_2_ (*M* = 381.12g/mol) 50.42% C and 9.52% H, found 50.17% C and 9.44% H.

### Synthesis of (*t*Bu_2_P)_3_B (**1c**)

To the suspension of 228 mg (1.5 mmol) of *t*Bu_2_PLi in 4 mL of toluene was added the solution of 0.4 mL (0.5 mmol, 0.8 M) of BBr_3_ in toluene at −30 °C. After being warmed to room temperature, the reaction mixture was stirred for 24 h, and toluene was evaporated under reduced pressure. The solid residue was dissolved in petroleum ether; LiBr was removed by filtration, and the solute was concentrated to a volume of 1.5 mL. Red crystals of **1c** (161 mg, 0.36 mmol, 72% yield) were isolated at −30 °C: ^1^H NMR (C_6_D_6_, 400 MHz, 298 K) δ 1.58 (broad m, overlapped 54H, (C**H**_3_)_3_C); ^11^B NMR (C_6_D_6_, 128 MHz, 298 K) δ 56.4 (broad s, (*t*Bu_2_P)_3_**B**); ^31^P{^1^H} NMR (C_6_D_6_, 162 MHz, 298 K) δ 40.8 (broad s *t*Bu_2_**P**B), 39.7 (s, (*t*Bu_2_**P**)_2_, 8.24%), 19.5 (s, *t*Bu_2_**P**H, 7.23%); ^13^C{^1^H} NMR (C_6_D_6_, 100 MHz, 298 K) δ 34.5 (m, overlapped, (**C**H_3_)_3_C), 37.5 (m, overlapped, (CH_3_)_3_**C**); elemental analysis calcd for C_24_H_54_BP_3_ (*M* = 446.42g/mol) 64.57% C and 12.19% H, found 63.72% C and 11. 91% H.

### Synthesis of (*t*Bu_2_P)_2_BPCy_2_ (**2c**)

To the suspension of 102 mg (0.5 mmol) of Cy_2_PLi in 2 mL of toluene was added the solution of 190 mg (0.5 mmol) of (*t*Bu_2_P)_2_BBr in 4 mL of toluene at −40 °C. After being warmed to room temperature, the reaction mixture was stirred for 72 h, and then toluene was evaporated under reduced pressure. The solid residue was dissolved in petroleum ether; LiBr was removed by filtration, and the solute was concentrated to a volume of 0.7 mL. Pale orange crystals of **2c** (197 mg, 0.395 mmol, 79% yield) were isolated at −30 °C. The synthesis in Et_2_O is shorter and takes <24 h: ^1^H NMR (C_6_D_6_, 400 MHz, 298 K) δ 1.24 (m, overlapped, 6H, C**H** (Cy)), 1.56 (m, broad, 36H, (C**H**_3_)_3_C), 1.66 (m, overlapped, 6H, C**H** (Cy)), 1.88 (m, overlapped, 4H, C**H** (Cy)), 2.04 (m, overlapped, 4H, C**H** (Cy)), 3.24 (m, overlapped, 2H, C**H** (Cy)); ^11^B NMR (C_6_D_6_, 128 MHz, 298 K) δ 50.9 (broad s, (*t*Bu_2_P)_2_**B**PCy_2_); ^31^P{^1^H} NMR (C_6_D_6_, 162 MHz, 298 K) δ 122.4 (broad s, Cy_2_**P**B), −1.7 (d, ^2^*J*_PP_ = 83 Hz, *t*Bu_2_**P**B); ^13^C{^1^H} NMR (C_6_D_6_, 100 MHz, 298 K) δ 25.7 (s, **C**H (Cy)), 27.9 (d, *J*_CP_ = 11 Hz, **C**H (Cy)), 33.2 (broad m, overlapped, (CH_3_)_3_**C**), 33.9 (m, overlapped, (**C**H_3_)_3_C), 34.0 (m, overlapped, **C**H (Cy)), 39.5 (m, overlapped, P**C**H (Cy)); elemental analysis calcd for C_28_H_58_BP_3_ (*M* = 498.49 g/mol) 67.46% C and 11.73% H, found 67.07% C and 11.55% H.

### Synthesis of (*t*Bu_2_P)_2_BP*i*Pr_2_ (**3c**)

To the suspension of 62 mg (0.5 mmol) of *i*Pr_2_PLi in 2 mL of toluene was added the solution of 190 mg (0.5 mmol) of (*t*Bu_2_P)_2_BBr in 3 mL of toluene at −40 °C. After being warmed to room temperature, the reaction mixture was stirred for 9 days, and toluene was evaporated under reduced pressure. The solid residue was dissolved in petroleum ether; LiBr was removed by filtration, and the solute was concentrated to a volume of 2 mL. Pale orange crystals of **3c** (186 mg, 0.444 mmol, 89% yield) were isolated at −30 °C. The synthesis can be accelerated in Et_2_O and is then almost immediate: ^1^H NMR (C_6_D_6_, 400 MHz, 298 K) δ 1.35 (dd, ^3^*J*_HH_ = 7 Hz, ^3^*J*_PH_ = 14 Hz, 12H, (C**H**_3_)_2_CH), 1.52 (d, ^3^*J*_HH_ = 11 Hz, 36H, (C**H**_3_)_3_C), 3.30 (m, 2H, (CH_3_)_2_C**H**); ^11^B NMR (C_6_D_6_, 128 MHz, 298 K) δ 50.7 (broad s, (*t*Bu_2_P)_2_**B**P*i*Pr_2_); ^31^P{^1^H} NMR (C_6_D_6_, 162 MHz, 298 K) δ 130.1 (broad s, *i*Pr_2_**P**B), −3.2 (d, ^2^*J*_PP_ = 88 Hz, *t*Bu_2_**P**B); ^13^C{^1^H} NMR (C_6_D_6_, 100 MHz, 298 K) δ 23.5 (d, ^2^*J*_CP_ = 6 Hz, (**C**H_3_)_2_CH), 28.6 (m, (CH_3_)_2_**C**H), 33.3 (m, (CH_3_)_3_**C**), 33.9 (m, (**C**H_3_)_3_C); elemental analysis calcd for C_22_H_50_BP_3_ (*M* = 418.36 g/mol) 63.16% C and 12.05% H, found 62.37% C and 11.85% H.

### Synthesis of (*t*Bu_2_P)_2_BP*t*BuPh (**4c**)

To the suspension of 86 mg (0.5 mmol) of *t*BuPhPLi in 4 mL of toluene was added the solution of 190 mg (0.5 mmol) of (*t*Bu_2_P)_2_BBr in 3 mL of toluene at −40 °C. After being warmed to room temperature, the reaction mixture was stirred for 9 days, and toluene was evaporated under reduced pressure. The solid residue was dissolved in petroleum ether; LiBr was removed by filtration, and the solute was concentrated to a volume of 2 mL. Red crystals of **4c** (175 mg, 0.375 mmol, 75% yield) were isolated at −30 °C. The synthesis in Et_2_O is shorter and takes <2 h: ^1^H NMR (C_6_D_6_, 400 MHz, 298 K) δ 1.48 (m, broad, 36H, (C**H**_3_)_3_C), 1.54 (d, ^3^*J*_HH_ = 15 Hz, 9H, (C**H**_3_)_3_CPPh), 7.02 (m, overlapped, 3H, C-**H**_**para**_ and C-**H**_**meta**_), 7.55 (t, ^3^*J*_HH_ = 8 Hz, 2H, C-**H**_**ortho**_); ^11^B NMR (C_6_D_6_, 128 MHz, 298 K) δ 59.7 (broad s, (*t*Bu_2_P)_2_**B**P*t*BuPh)); ^31^P{^1^H} NMR (C_6_D_6_, 162 MHz, 298 K) δ 86.2 (broad s, *t*BuPh**P**B), 19.6 (s, *t*Bu_2_**P**H, 1.9%), 12.4 (broad d, ^2^*J*_PP_ = 106 Hz (*t*Bu_2_**P**B)), −5.6 (s, *t*BuPh**P**H, 2.6%); ^13^C{^1^H} NMR (C_6_D_6_, 100 MHz, 298 K) δ 33.8 (m, overlapped, (**C**H_3_)_3_CPPh), 34.0 (m, overlapped, (**C**H_3_)_3_C), 34.7 (dd, ^2^*J*_CP_ = 10 Hz, ^1^*J*_CP_ = 19 Hz, (CH_3_)_3_**C**), 38.9 (broad d, ^2^*J*_CP_ = 10 Hz, (CH_3_)_3_**C**), 127.3 (d, **C**_**p**_, ^4^*J*_CP_ = 9 Hz), 129.2 (d, ^3^*J*_CP_ = 2 Hz, **C**_**m**_), 135.5 (m, **C**_**i**_), 138.2 (d, ^2^*J*_CP_ = 7 Hz, **C**_**o**_); elemental analysis calcd for C_26_H_50_BP_3_ (*M* = 466.41 g/mol) 66.95% C and 10.80% H, found 66.03% C and 10.54% H.

### Synthesis of (*t*Bu_2_P)_2_BPPh_2_ (**5c**)

To the suspension of 96 mg (0.5 mmol) of Ph_2_PLi in 2 mL of toluene was added the solution of 190 mg (0.5 mmol) of (*t*Bu_2_P)_2_BBr in 3 mL of toluene at −40 °C. After being warmed to room temperature, the reaction mixture was stirred for 30 min, and then toluene was evaporated under reduced pressure. The solid residue was dissolved in petroleum ether; LiBr was removed by filtration, and the solute was concentrated to a volume of 2 mL. Large red crystals of **5c** (189 mg, 0.388 mmol, 78% yield) were isolated at −30 °C: ^1^H NMR (C_6_D_6_, 400 MHz, 298 K) δ 1.46 (m, broad, 36H, (C**H**_3_)_3_C), 7.00 (d, ^3^*J*_HH_ = 7 Hz, 2H, C-**H**_**para**_), 7.06 (t, ^3^*J*_HH_ = 7 Hz, 4H, C-**H**_**meta**_), 7.65 (t, ^3^*J*_HH_ = 7 Hz, 4H, C-**H**_**ortho**_); ^11^B NMR (C_6_D_6_, 128 MHz, 298 K) δ 64.0 (broad s, (*t*Bu_2_P)_2_**B**PPh_2_); ^31^P{^1^H} NMR (C_6_D_6_, 162 MHz, 298 K) δ 52.3 (broad d, ^2^*J*_PP_ = 98 Hz, *t*Bu_2_**P**B), 19.5 (s, *t*Bu_2_**P**H, 7%), −12.4 (broad t, ^2^*J*_PP_ = 98 Hz, Ph_2_**P**B), −40.7 (s, Ph_2_**P**H, 4.4%); ^13^C{^1^H} NMR (C_6_D_6_, 100 MHz, 298 K) δ 33.9 (m, overlapped, (**C**H_3_)_3_C), 36.9 (broad s, overlapped, (CH_3_)_3_**C**), 127.6* (s, **C**_**p**_), 128.1 (d, ^3^*J*_CP_ = 8 Hz, **C**_**m**_), 136.2 (d, ^2^*J*_CP_ = 16 Hz, **C**_**o**_), 139.4 (t, ^1^*J*_CP_ = 8 Hz, **C**_**i**_) (*on the basis of 135DEPT NMR); elemental analysis calcd for C_28_H_46_BP_3_ (*M* = 486.40 g/mol) 69.14% C and 9.53% H, found 68.75% C and 9.40% H.

### Reaction of BBr_3_ with *t*BuPhPLi in a 1:3 Molar Ratio

To the suspension of 255 mg (1.5 mmol) of *t*BuPhPLi in 4 mL of toluene was added the solution of 0.625 mL (0.5 mmol, 0.8 M) of BBr_3_ in toluene at −30 °C. After being warmed to room temperature, the reaction mixture was stirred for 6 days. Then the solvent was evaporated under reduced pressure, and the solid residue was partially dissolved in petroleum ether. LiBr was removed by filtration, and the solute was concentrated to a volume of 2 mL. The residue that was not soluble in petroleum ether was redissolved in toluene. Crystals of **4c′** suitable for X-ray diffraction analysis were isolated at −30 °C from the concentrated petroleum ether fraction. Regardless of the reaction stoichiometry, **4a** is always the main product.

### Synthesis of **3d**

To the solution of 84 mg (0.2 mmol) of **3c** in 2 mL of toluene was added the solution of 0.52 mL (0.4 mmol, 0.8 M) of BH_3_·SMe_2_ in toluene at −30 °C. After being warmed to room temperature, the reaction mixture was stirred for 24 h. Then the solvent was evaporated under reduced pressure, the solid residue redissolved in petroleum ether, and the solute concentrated to a volume of 1.5 mL. Colorless crystals of **3d** (64 mg, 0.143 mmol, 72% yield) were isolated at −30 °C: ^1^H NMR (C_6_D_6_, 400 MHz, 298 K) δ 0.84–2.15 (very broad signals, overlapped, 5H, B**H**_2_ and B**H**_3_), 0.94 (dd, ^3^*J*_HH_ = 7 Hz, ^3^*J*_PH_ = 14 Hz, 3H, (C**H**_3_)_2_CH), 1.17 (dd, ^3^*J*_HH_ = 7 Hz, ^3^*J*_PH_ = 12 Hz, 3H, (C**H**_3_)_2_CH), 1.22 (dd, ^3^*J*_HH_ = 7 Hz, ^3^*J*_PH_ = 15 Hz, 3H, (C**H**_3_)_2_CH), 1.31 (dd, ^3^*J*_HH_ = 7 Hz, ^3^*J*_PH_ = 15 Hz, 3H, (C**H**_3_)_2_CH), 1.37 (broad d, 18H, (C**H**_3_)_3_C), 1.41 (dd, ^3^*J*_HH_ = 8 Hz, ^3^*J*_PH_ = 13 Hz, 18H, (C**H**_3_)_3_C), 2.20 (m, 1H, (CH_3_)_2_C**H**), 2.36 (m, 1H, (CH_3_)_2_C**H**), 3.37 (very broad d, 1H, B1**H**); ^11^B NMR (C_6_D_6_, 128 MHz, 298 K) δ −23.9 (broad quintet, ^1^*J*_BP_ = 83 Hz, **B**H), −33.8 to −38.2 (two overlapped m, **B**H_2_ and **B**H_3_); ^31^P{^1^H} NMR (C_6_D_6_, 162 MHz, 298 K) δ 36.2 (broad s, *t*Bu_2_**P**B), 29.5 (broad s, *t*Bu_2_**P**B), 4.7 (broad s, *i*Pr_2_**P**B); ^13^C{^1^H} NMR (C_6_D_6_, 100 MHz, 298 K) δ 18.2 (d, ^2^*J*_CP_ = 4 Hz, (**C**H_3_)_2_CH), 18.6 (broad s, (**C**H_3_)_2_CH), 19.3 (broad d, ^2^*J*_CP_ = 2 Hz, (**C**H_3_)_2_CH), 21.1 (dd, ^1^*J*_CP_ = 19 Hz, ^2^*J*_CP_ = 4 Hz, (CH_3_)_2_**C**H), 21.4 (broad d, ^2^*J*_CP_ = 2 Hz, (**C**H_3_)_2_CH), 22.4 (dd, ^1^*J*_CP_ = 19 Hz, ^2^*J*_CP_ = 4 Hz, (CH_3_)_2_**C**H), 22.9 (m, (CH_3_)_2_**C**H), 29.8 (broad s, (**C**H_3_)_3_C), 30.0 (broad d, ^2^*J*_CP_ = 2 Hz, (**C**H_3_)_3_C), 30.1 (broad d, ^2^*J*_CP_ = 3 Hz, (**C**H_3_)_3_C), 30.7 (broad d, ^2^*J*_CP_ = 2 Hz, (**C**H_3_)_3_C), 32.4 (m, overlapped, (CH_3_)_3_**C**), 35.8 (dd, ^1^*J*_CP_ = 23 Hz, ^2^*J*_CP_ = 14 Hz, (CH_3_)_3_**C**); elemental analysis calcd for C_22_H_56_B_3_P_3_ (*M* = 446.03 g/mol) 59.24% C and 12.65% H, found 58.46% C and 12.63% H.

## Supporting Information Available

Crystallographic details, NMR spectroscopic details, and computational details (PDF)

## Accession Codes

CCDC 2114349–2114360 contain the supplementary crystallographic data for this paper. These data can be obtained free of charge via www.ccdc.cam.ac.uk/data_request/cif, or by emailing data_request@ccdc.cam.ac.uk, or by contacting The Cambridge Crystallographic Data Centre, 12 Union Road, Cambridge CB2 1EZ, UK; fax: +44 1223 336033.
