# Supplementary material for: Monomeric Triphosphinoboranes: Intramolecular Lewis Acid–Base Interactions between Boron and Phosphorus Atoms
Source: Inorg Chem. 2022 Feb 28;61(10):4361–70. doi: 10.1021/acs.inorgchem.1c03618 (PMC8924927; doi:10.1021/acs.inorgchem.1c03618)
Supplement: Supplementary file 1 — ic1c03618_si_001.pdf [file ic1c03618_si_001.pdf]

Monomeric triphosphinoboranes: Intramolecular Lewis acid-base interactions between boron and phosphorus atoms

Anna Ordyszewska, Natalia Szynkiewicz, Jarosław Chojnacki, Rafał Grubba*

Department of Inorganic Chemistry, Faculty of Chemistry, Gdańsk University of Technology, G. Narutowicza St. 11/12. PL-80-233, Gdansk, Poland

*rafal.grubba@pg.edu.pl

1. Crystallographic Details
2. NMR Spectroscopic Details
3. Computational Details
4. References

1. Crystallographic Details

Diffraction intensity data for all crystals were collected on an IPDS 2T dual beam diffractometer (STOE & Cie GmbH, Darmstadt, Germany) at 120.0(2) K with MoKa radiation of a microfocus X-ray source (GeniX 3D Mo High Flux, Xenocs, Sassenage, 50 kV, 1.0 mA, and λ = 0.71069 Å). Investigated crystals were thermostated under a nitrogen stream at 120 K or 130 K using the CryoStream-800 device (Oxford CryoSystem, UK) during the entire experiment.

Data collection and data reduction were controlled by using the X-Area 1.75 program (STOE, 2015). Absorption correction was performed only for data with absorption coefficient > 0.5 mm^{-1}. The structures were solved using intrinsic phasing implemented in SHELXT and refined anisotropically using the program packages Olex2[1] and SHELX-2015[2,3]. Positions of hydrogen atoms were calculated geometrically taking into account isotropic temperature factors. All H-atoms were refined as riding on their parent atoms with the usual restraints.

Special treatment for selected structures was applied. Structure **1b** was refined as an inversion twin, with domain fractions of 0.055(10) and 0.945(10). Absorption correction, by the integration method, was applied. Structure **1c** contained regions of strongly disordered solvent (petroleum ether). Electron density from the regions (four locations, with ca 65e^- and volume 385 $Å^3$ each, are reported by the program) were removed using the SQUEEZE procedure implemented in OLEX2 environment. Relatively high residual electron density most likely stems from partial occupation of the same position by molecule of inverted triphosphinoborane. It is not reliable to model inversion of the whole molecule since only heavy atoms (P) give noticeable electron density. We also obtained a solvent free structure of the compound, but due to its bad quality we will not discuss it here. Structure **2a**, due to high bromine content, required numerical absorption correction of hkl intensities. Structure **2c** was refined with the two $Pt\mathrm{Bu}_2$ groups disordered in two positions with almost equal population. Structure **3a** required absorption correction and was refined as the non-merohedral twin with domain mass fractions of 0.372(3) and 0.628(3). Structure **3c** was refined without any special treatment, just two reflections were omitted as being affected by the beamstop. Hydrogen B-H atoms for **3d** were found in the Fourier electron density map and were refined without constrants. Triphosphinoboranes **4c**, **4c'** and **5c** were solved and refined without special tereatment. Structures **4a** and **5a** required absorption correction (numerical). Additionally, best results were obtained by refinement of **5a** as a two component twin with the twin law [{0.5 0 -0.5} {0 -1 0} {-1.5 0 -0.5}] and domain mass fractions of 0.0155(8) and 0.9845(8).

Crystallographic data for all structures reported in this paper have been deposited with the Cambridge Crystallographic Data Centre as supplementary publication Nos. CCDC 2114349-2114360. The data can be obtained free of charge from The Cambridge Crystallographic Data Centre via www.ccdc.cam.ac.uk/structures.

Table S1. Crystal data and structure refinement for **2a**, **3a**, **4a**, and **5a**.

	**2a**	**3a**	**4a**	**5a**
CCDC no.	2114351	2114353	2114356	2114359
Chemical formula	$C_{24}H_{44}B_2Br_4P_2$	$C_{12}H_{28}B_2Br_4P_2$	$C_{20}H_{28}B_2Br_4P_2$	$C_{24}H_{20}B_2Br_4P_2$
M_r (g/mol)	735.79	575.54	671.62	711.60
Temperature (K)	120	120	130	120
Radiation type	Mo $K\alpha$	Mo $K\alpha$	Mo $K\alpha$	Mo $K\alpha$

Crystal system	Monoclinic	Monoclinic	Monoclinic	Monoclinic
Space group	$P2_1/c$	$P2_1/c$	$P12_1/m1$	$P2_1/n$
a (Å)	17.8138 (17)	8.695 (3)	7.9958 (4)	12.0213 (9)
b (Å)	19.3115 (14)	16.955 (7)	13.1263 (6)	13.1892 (7)
c (Å)	17.585 (2)	13.889 (5)	12.2343 (7)	17.2246 (9)
α (°)	90	90	90	90
β (°)	92.569 (9)	96.52 (3)	104.357 (5)	109.845 (5)
γ (°)	90	90	90	90
V (Å^3)	6043.3 (10)	2034.4 (12)	1243.95 (11)	2568.8 (3)
Z	8	4	2	4
D_x (g/cm^3)	1.617	1.879	1.793	1.840
Crystal size (mm)	0.31 × 0.27 × 0.06	0.34 × 0.07 × 0.03	0.11 × 0.07 × 0.04	0.28 × 0.22 × 0.04
θ Range (°)	2.3–29.6	2.4-29.3	2.3-29.2	2.4-25.9
Reflections collected/unique	62867/16267	5482/5482	18154/3485	25375/4990
Completness to θmax (%)	96.0	98.8	99.4	100
Data/restraints/parameters	16267/0/577	5482/0/190	3485/0/146	4990/0/290
Goodness-of-fit on F^2	1.029	1.056	1.044	1.078
Final R indexes [I>2σ(I)]<br>R indexes (all data)	R_1 = 0.338<br>R_2 = 0.0503<br>wR_1 = 0.0715<br>wR_2 = 0.0785	R_1 = 0.0532,<br>R_2 = 0.0917,<br>wR_1 = 0.1372,<br>wR_2 = 0.1613	R_1 = 0.0209,<br>R_2 = 0.0269,<br>wR_1 = 0.0467,<br>wR_2 = 0.0487	R_1 = 0.0851,<br>R_2 = 0.1007,<br>wR_1 = 0.2177,<br>wR_2 = 0.2350
Largest diff. peak and hole (e Å^{-3})	0.619, -0.613	0.980, -1.494	0.439, -0.341	1.767, -1.956

Table S2. Crystal data and structure refinement for **1b**, **1c**, **2c**, and **3c**.

	**1b**	**1c**	**2c**	**3c**
CCDC no.	2114349	2114350	2114352	2114354
Chemical formula	$C_{16}H_{36}BBrP_2$	$C_{24}H_{54}BP_3$	$C_{28}H_{58}BP_3$	$C_{22}H_{50}BP_3$
M_r (g/mol)	381.11	446.39	498.46	418.34
Temperature (K)	120	120	130	120
Radiation type	Mo $K\alpha$	Mo $K\alpha$	Mo $K\alpha$	Mo $K\alpha$
Crystal system	Orthorombic	Trigonal	Monoclinic	Triclinic
Space group	$P2_12_12_1$	$P31c$	$P2_1/c$	$P\overline{1}$
a (Å)	8.7098 (10)	30.923 (2)	15.0157 (5)	9.5356 (4)
b (Å)	8.7668 (7)	30.923 (2)	10.4187 (3)	10.3051 (5)
c (Å)	26.894 (2)	8.0230 (6)	21.1082 (6)	14.6242 (7)
α (°)	90	90	90	83.214 (4)
β (°)	90	90	110.281 (2)	82.521 (4)
γ (°)	90	120	90	66.308 (3)
V (Å^3)	2053.5 (3)	6643.8 (11)	3097.53 (17)	1301.30 (11)
Z	4	8	4	2
D_x (g/cm^3)	1.233	0.893	1.069	1.068

Crystal size (mm)	0.41×0.28×0.19	0.24 × 0.12 × 0.11	0.26 × 0.14 × 0.06	0.13 × 0.11 × 0.05
θ Range (°)	2.4-29.5	2.3-29.3	2.2-29.3	2.3–29.7
Reflections collected/unique	31476/5524	101259/11988	20254/8339	19893/6984
Completness to θmax (%)	99.7	99.1	98.5	99.3
Data/restraints/parameters	5524/0/194	11988/1/361	8339/0/477	6984/0/251
Goodness-of-fit on F^2	1.061	1.370	1.108	1.046
Final R indexes [I>2σ(I)]<br><br>R indexes (all data)	R_1 = 0.0376<br>R_2 = 0.0508<br>wR_1 = 0.0836<br>wR_2 = 0.078	R_1 = 0.1212<br>R_2 = 0.1544,<br>wR_1 = 0.3075,<br>wR_2 = 0.3521	R_1 = 0.0597,<br>R_2 =0.0838,<br>wR_1 = 0.1294,<br>wR_2 = 0.1372	R_1 = 0.0330,<br>R_2 = 0.0446,<br>wR_1 = 0.0828,<br>wR_2 = 0.0870
Largest diff. peak and hole (e Å^{-3})	0.522, -0.314	3.284, -3.404	0.750, -0.371	0.371, -0.254

Table S3. Crystal data and structure refinement for **4c**, **4c'**, **5c**, and **3d**.

	**4c**	**4c'**	**5c**	**3d**
CCDC no.	2114357	2114358	2114360	2114355
Chemical formula	$C_{26}H_{50}BP_3$	$C_{30}H_{42}BP_3$	$C_{28}H_{46}BP_3$	$C_{22}H_{55}B_3P_3$
M_r (g/mol)	466.38	506.35	486.37	446.00
Temperature (K)	120	120	120	130
Radiation type	Mo Kα	Mo Kα	Mo Kα	Mo *K*α
Crystal system	Monoclinic	Monoclinic	Monoclinic	Monoclinic
Space group	$P2_1/n$	$P2_1/n$	$P2_1/n$	$P2_1/c$
a (Å)	9.2124 (2)	8.8077 (7)	10.9825 (11)	17.1580 (9)
b (Å)	19.1644 (4)	18.8477 (17)	8.7579 (7)	10.9343 (4)
c (Å)	16.1194 (4)	18.1291 (15)	30.770 (3)	15.3679 (9)
α (°)	90	90	90	90
β (°)	98.267 (2)	91.719 (7)	99.080 (8)	97.674 (4)
γ (°)	90	90	90	90
V (Å^3)	2816.31 (11)	3008.2 (4)	2922.4 (5)	2857.4 (2)
Z	4	4	4	4
D_x (g/cm^3)	1.100	1.118	1.105	1.034
Crystal size (mm)	0.23 × 0.12 × 0.09	0.39 × 0.19 × 0.12	0.31 × 0.23 × 0.03	0.23 × 0.11 × 0.09
θ Range (°)	2.4-29.2	2.2-29.3	2.4-29.2	2.3-29.3
Reflections collected/unique	28989/7593	44275/8109	35674/7855	14860/7670
Completness to θmax (%)	99.5	99.1	99.0	98.6
Data/restraints/parameters	7593/0/286	8109/0/316	7855/0/301	7670/0/289
Goodness-of-fit on F^2	1.034	1.053	1.057	1.035

Final R indexes [I>2σ(I)]<br><br>R indexes (all data)	R_1 = 0.0331,<br>R_2 = 0.0439,<br>wR_1 = 0.0822,<br>wR_2 = 0.0871	R_1 = 0.479,<br>R_2 = 0.641,<br>wR_1 =0.1250,<br>wR_2 = 0.1340	R_1 = 0.0408,<br>R_2 = 0.0601,<br>wR_1 = 0.0974,<br>wR_2 = 0.1055	R_1 = 0.0733,<br>R_2 = 0.1064,<br>wR_1 = 0.1930,<br>wR_2 = 0.2120
Largest difference peak and hole (e Å^{-3})	0.384, -0.201	0.690, -0.395	0.360, -0.375	0.922, -0.470

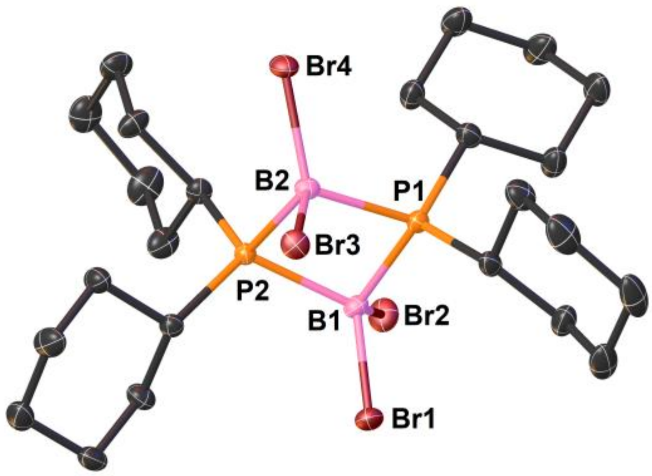

Figure S1. X-ray structure of **2a** showing the atom-numbering scheme. Ellipsoids are shown at 50% probability. H atoms have been omitted for clarity.

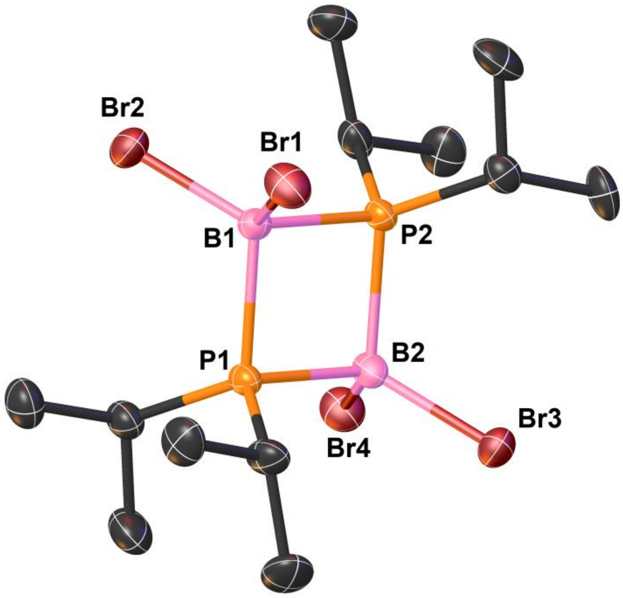

Figure S2. X-ray structure of **3a** showing the atom-numbering scheme. Ellipsoids are shown at 50% probability. H atoms have been omitted for clarity.

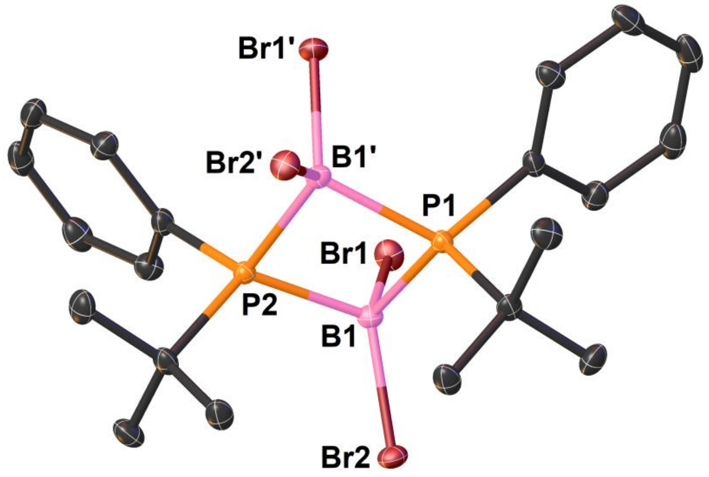

Figure S3. X-ray structure of **4a** showing the atom-numbering scheme. Ellipsoids are shown at 50% probability. H atoms have been omitted for clarity.

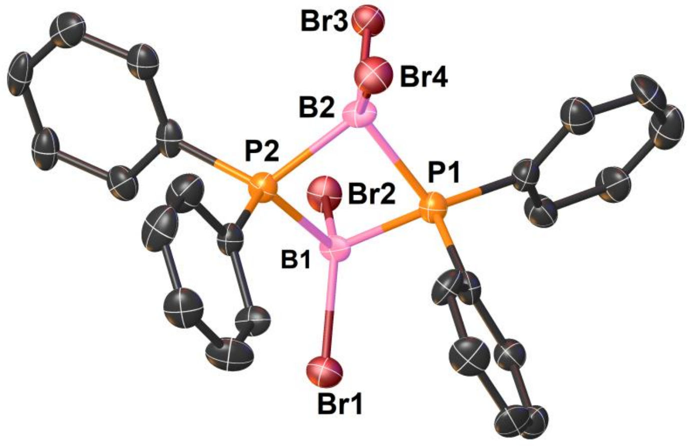

Figure S4. X-ray structure of **5a** showing the atom-numbering scheme. Ellipsoids are shown at 50% probability. H atoms have been omitted for clarity.

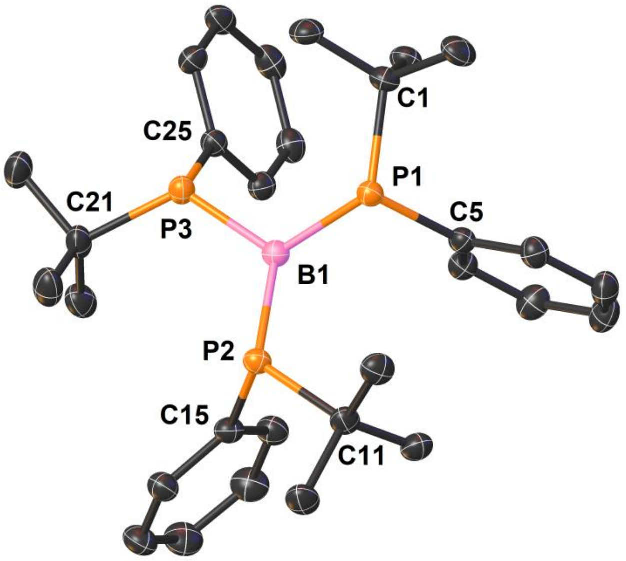

Figure S5. X-ray structure of **4c'** showing the atom-numbering scheme. Ellipsoids are shown at 50% probability. H atoms have been omitted for clarity.

2. NMR Spectroscopic Details

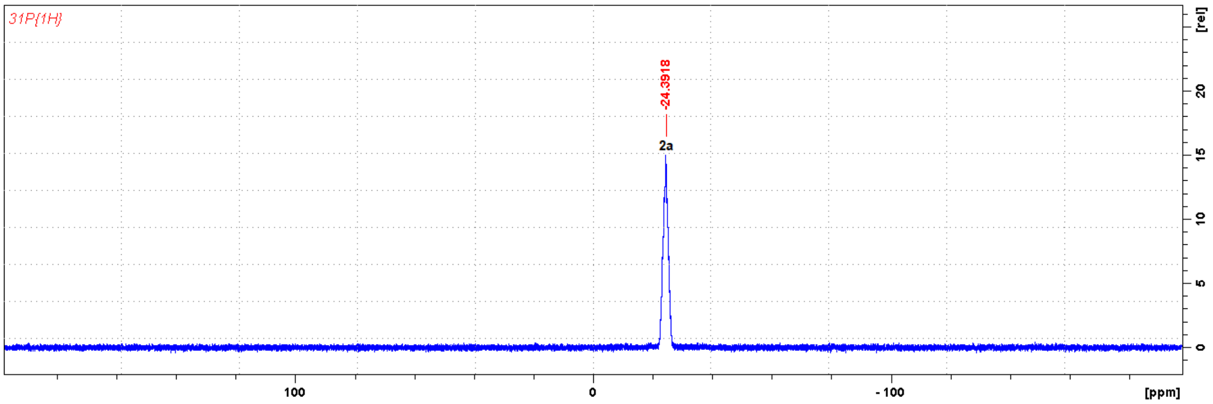

Figure S6. $^{31}P\{^{1}H\}$ NMR (C_6D_6, 162 MHz) spectrum of **2a**.

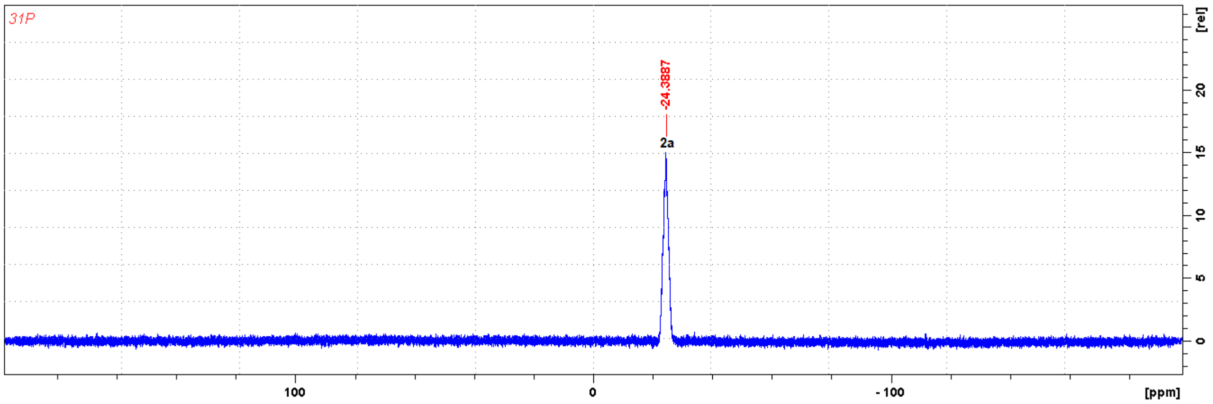

Figure S7. ^{31}P NMR (C_6D_6, 162 MHz) spectrum of **2a**.

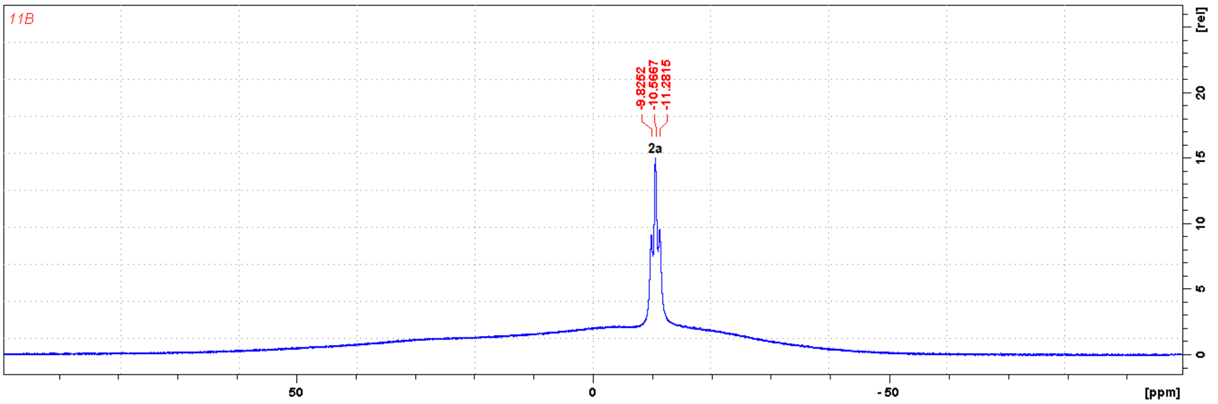

Figure S8. ^{11}B NMR (C_6D_6, 128 MHz) spectrum of **2a**.

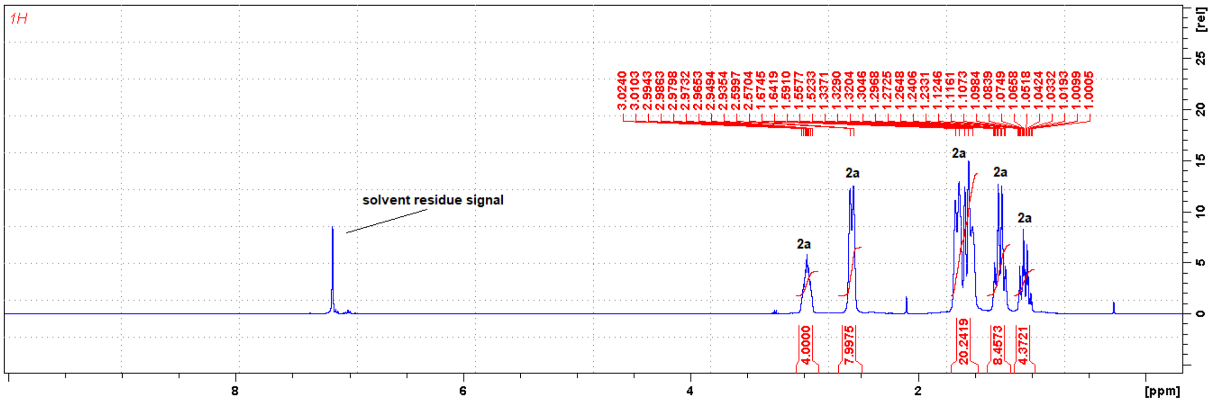

Figure S9. ^{1}H NMR (C_6D_6, 400 MHz) spectrum of **2a**.

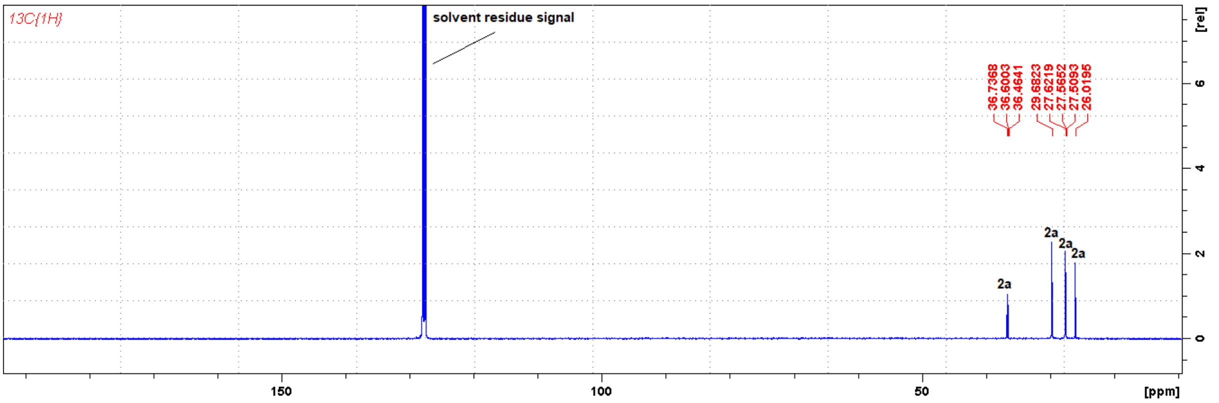

Figure S10. $^{13}C\{^{1}H\}$ NMR (C_6D_6, 100 MHz) spectrum of **2a**.

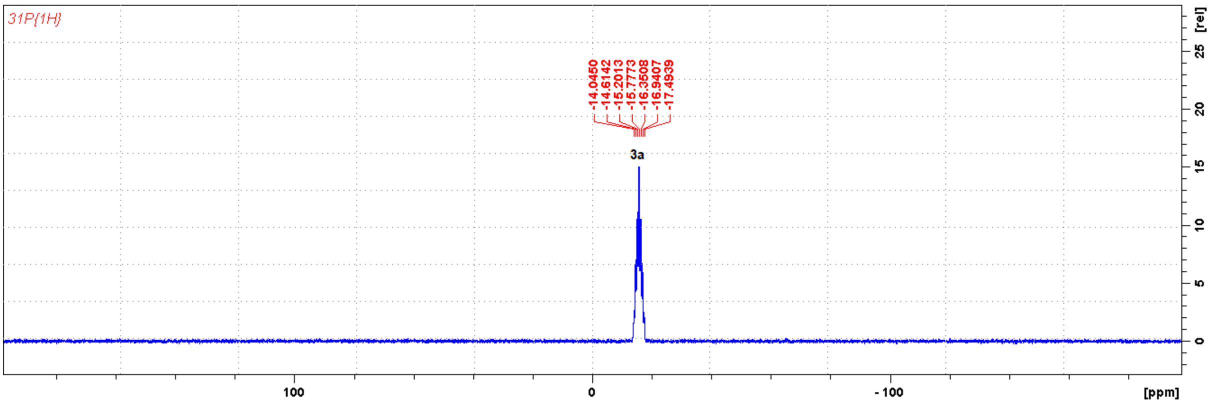

Figure S11. $^{31}P\{^{1}H\}$ NMR (C_6D_6, 162 MHz) spectrum of **3a**.

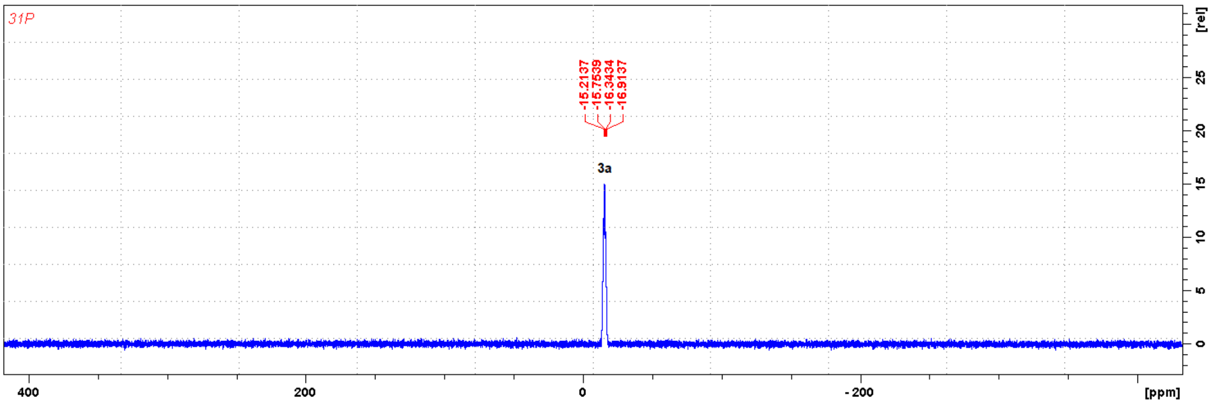

Figure S12. ^{31}P NMR (C_6D_6, 162 MHz) spectrum of **3a**.

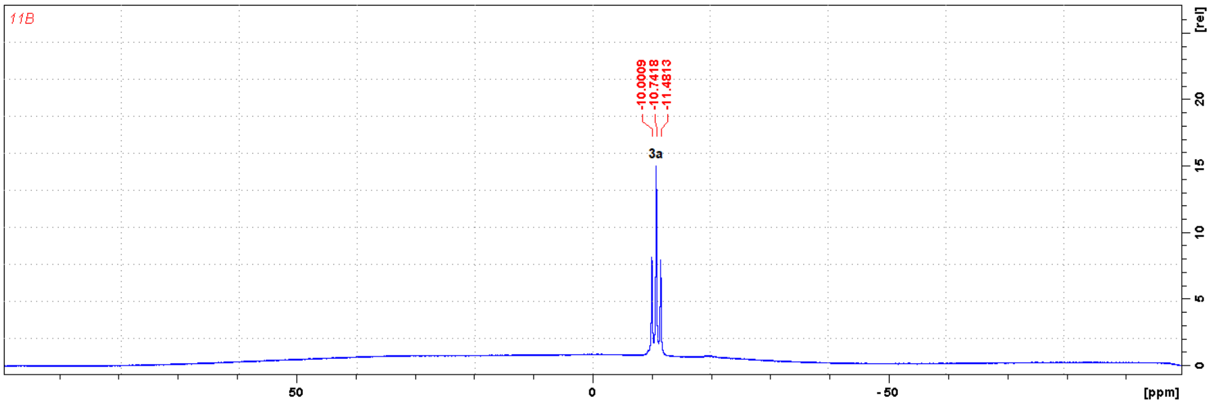

Figure S13. ^{11}B NMR (C_6D_6, 128 MHz) spectrum of **3a**.

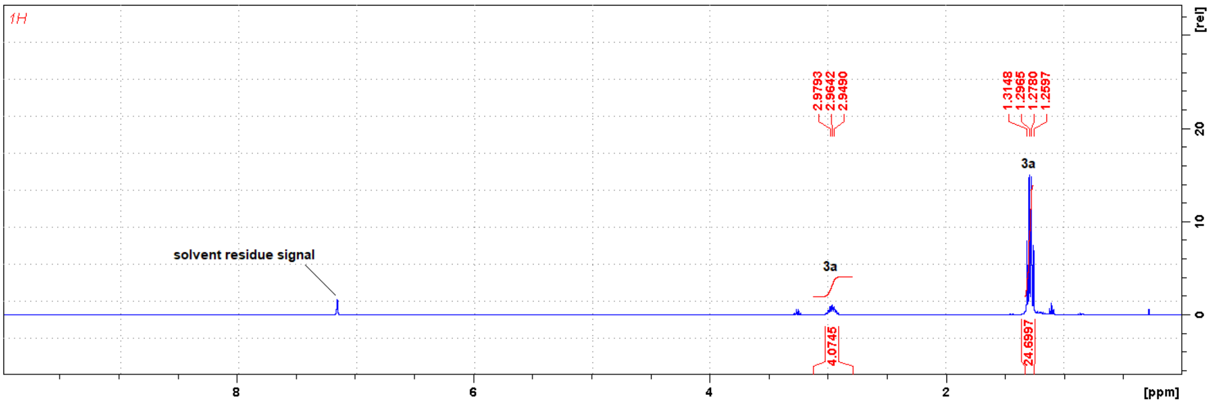

Figure S14. ^{1}H NMR (C_6D_6, 400 MHz) spectrum of **3a**.

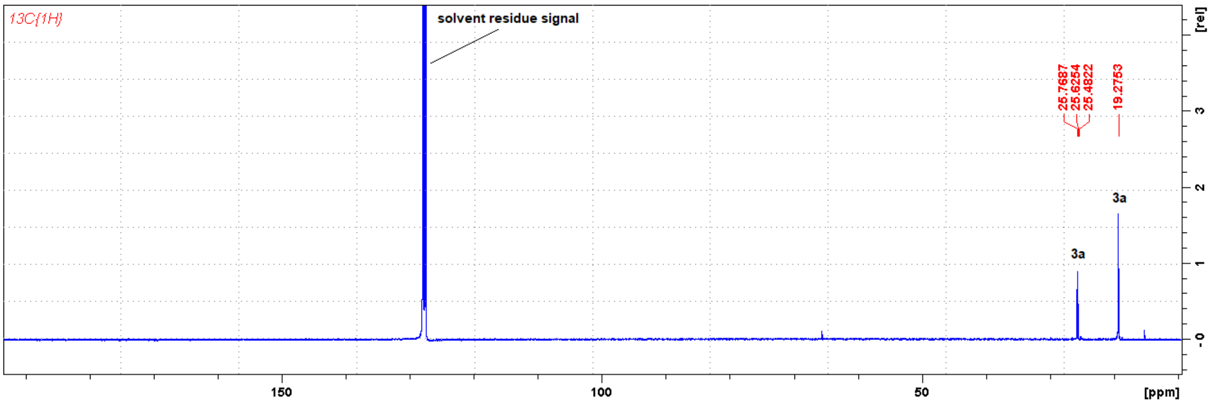

Figure S15. $^{13}C\{^{1}H\}$ NMR (C_6D_6, 100 MHz) spectrum of **3a**.

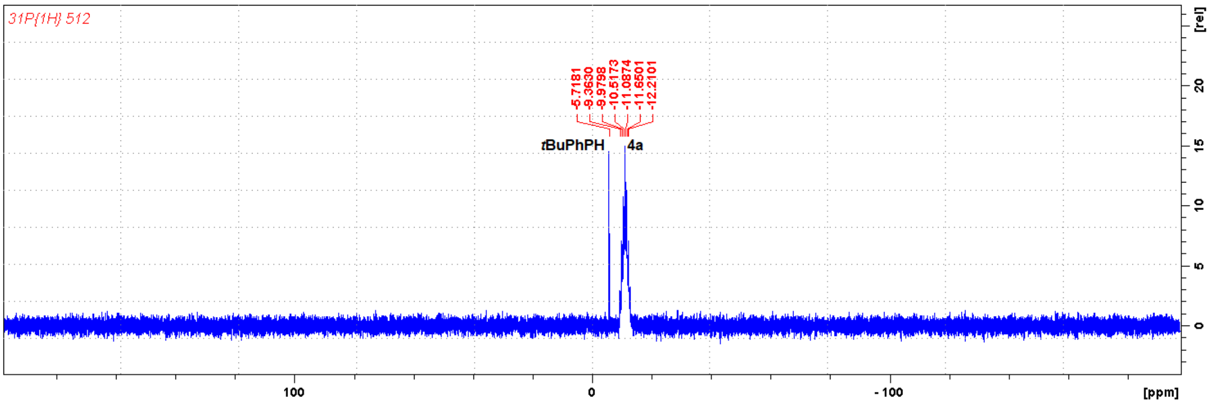

Figure S16. $^{31}P\{^{1}H\}$ NMR (C_6D_6, 162 MHz) spectrum of **4a**.

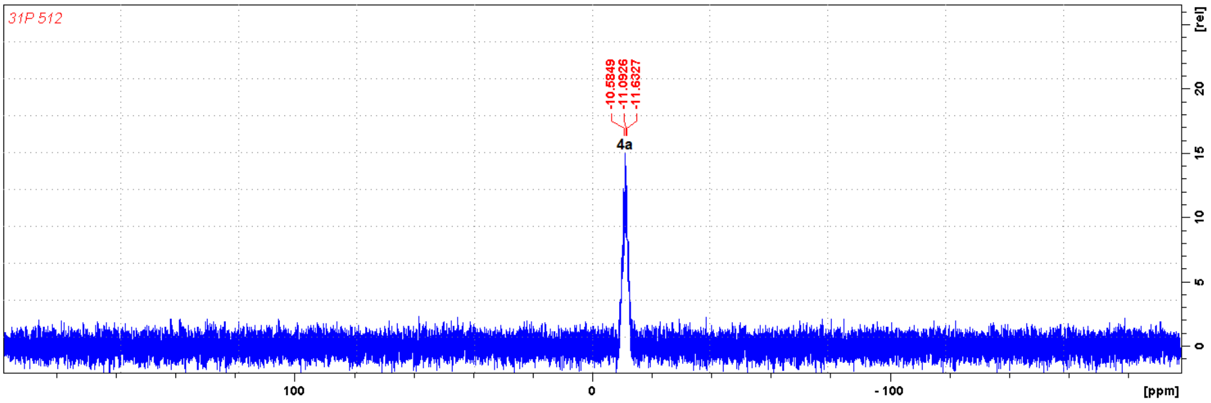

Figure S17. ^{31}P NMR (C_6D_6, 162 MHz) spectrum of **4a**.

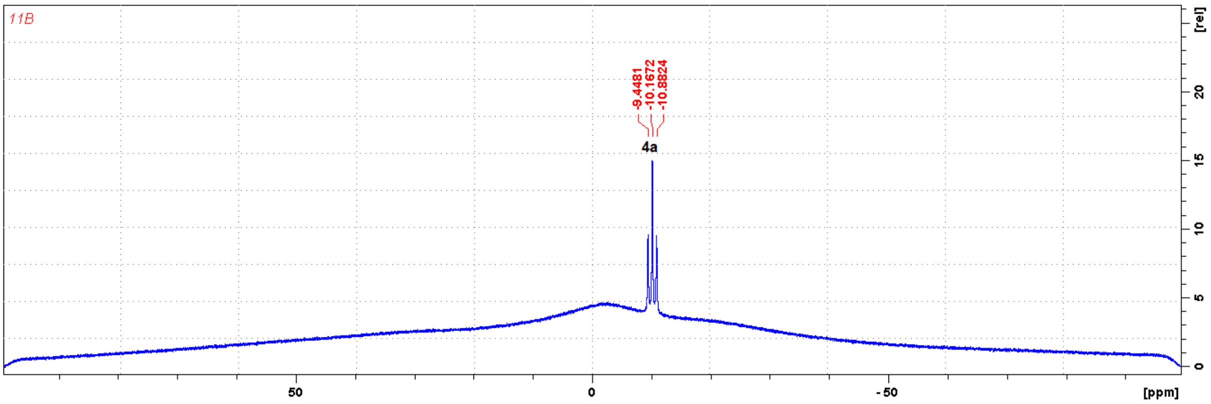

Figure S18. ^{11}B NMR (C_6D_6, 128 MHz) spectrum of **4a**.

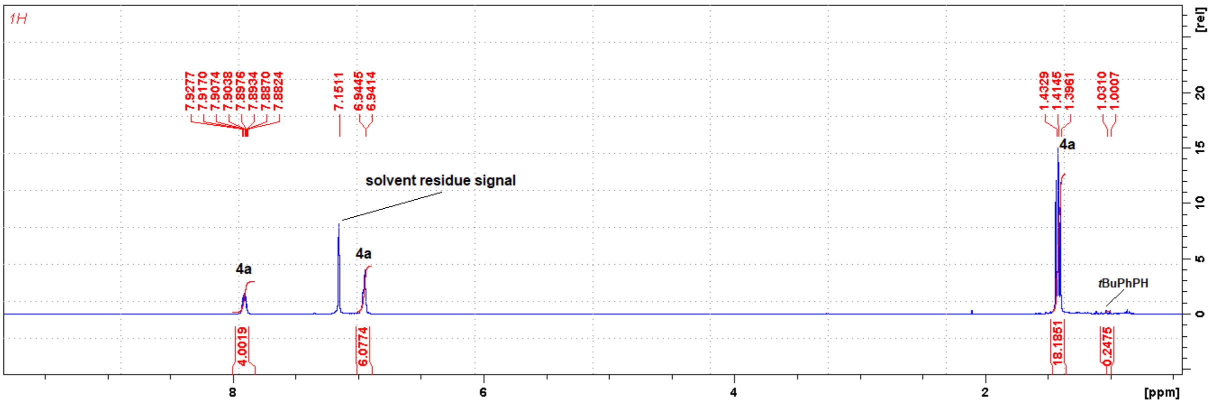

Figure S19. ^{1}H NMR (C_6D_6, 400 MHz) spectrum of **4a**.

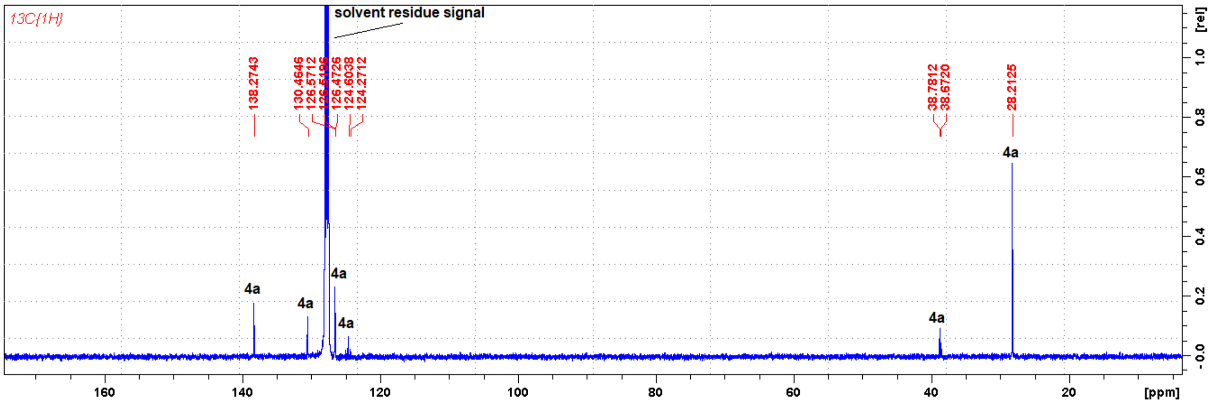

Figure S20. $^{13}C\{^1H\}$ NMR (C_6D_6, 100 MHz) spectrum of **4a**.

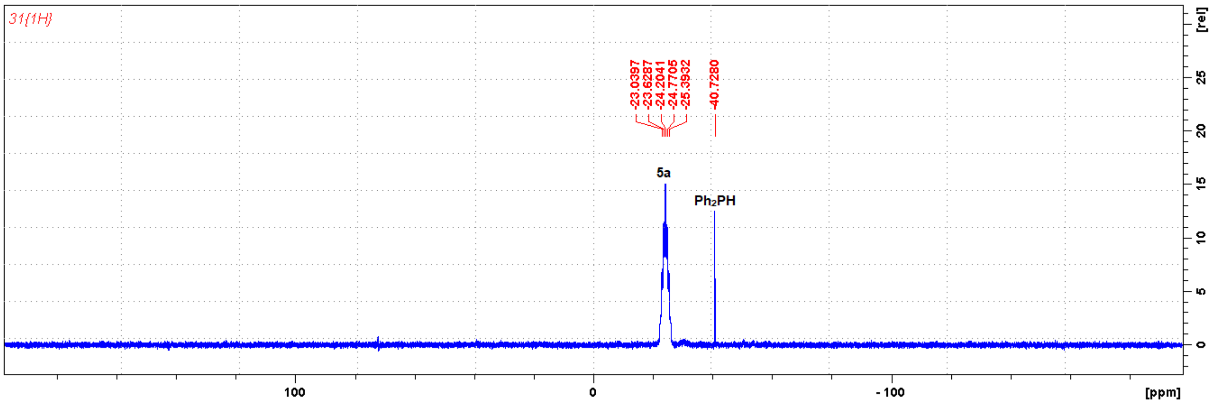

Figure S21. $^{31}P\{^1H\}$ NMR (C_6D_6, 162 MHz) spectrum of **5a**.

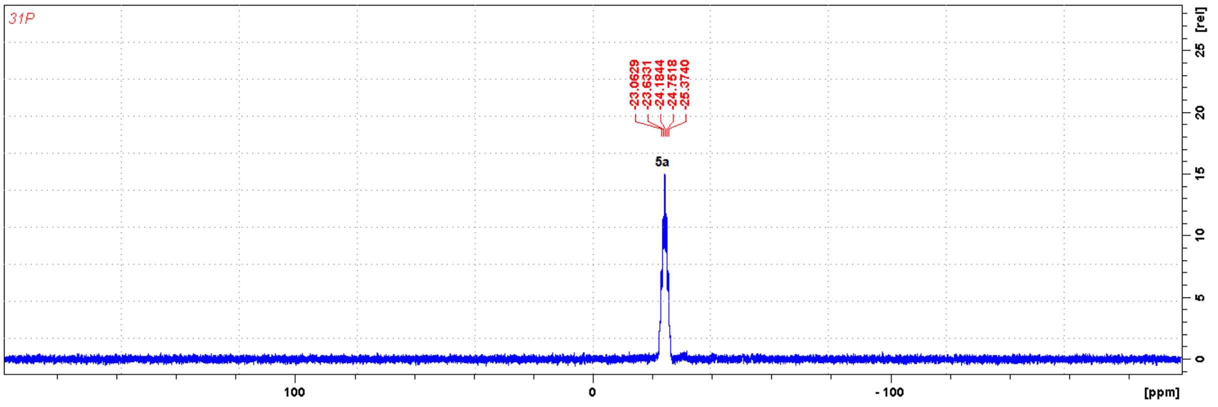

Figure S22. ^{31}P NMR (C_6D_6, 162 MHz) spectrum of **5a**.

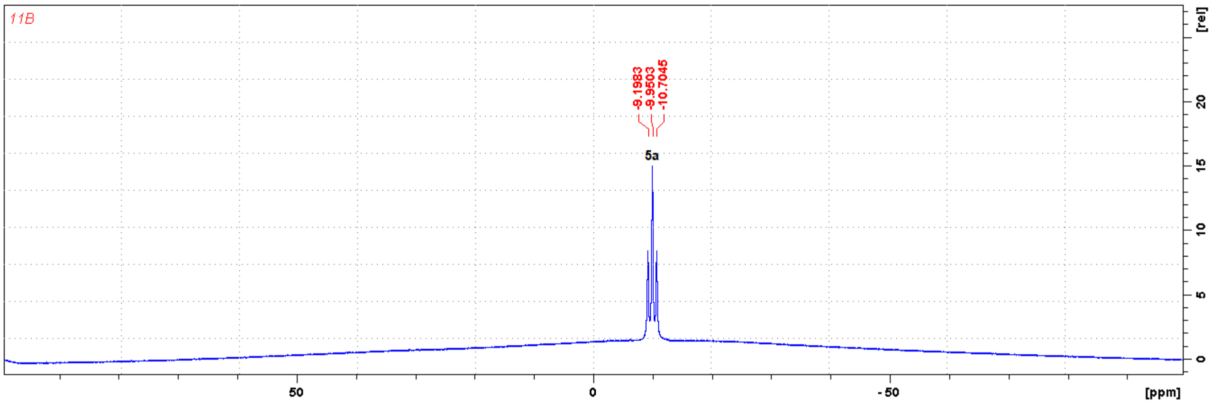

Figure S23. ^{11}B NMR (C_6D_6, 128 MHz) spectrum of **5a**.

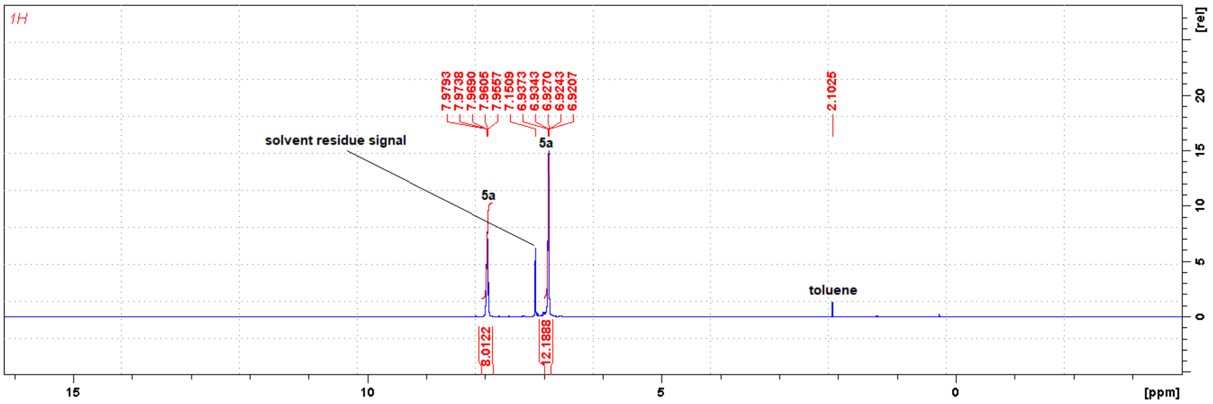

Figure S24. ^{1}H NMR (C_6D_6, 400 MHz) spectrum of **5a**.

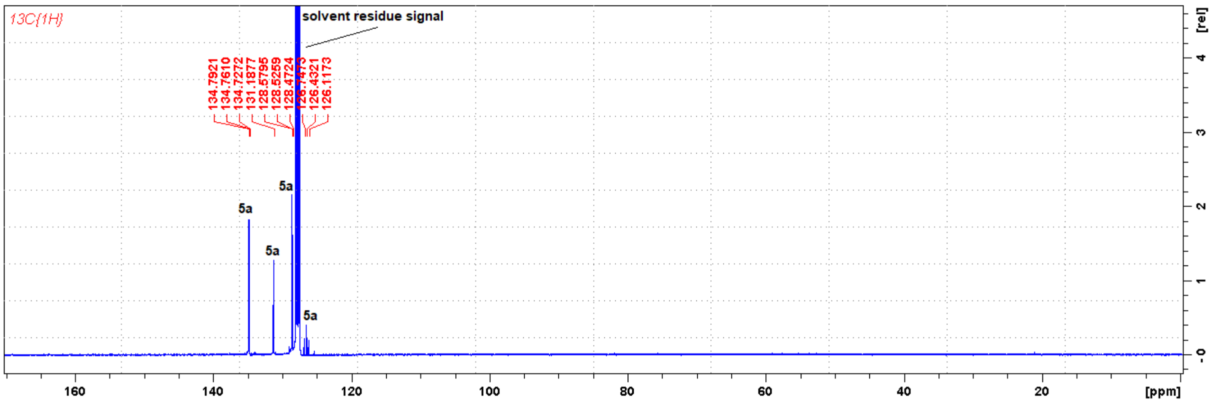

Figure S25. $^{13}C\{^{1}H\}$ NMR (C_6D_6, 100 MHz) spectrum of **5a**.

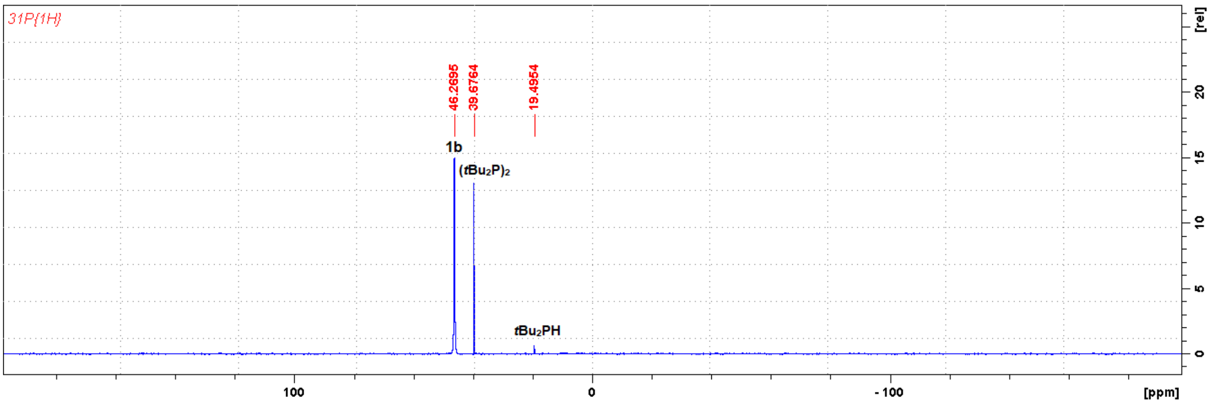

Figure S26. $^{31}P\{^1H\}$ NMR (C_6D_6, 162 MHz) spectrum of **1b**.

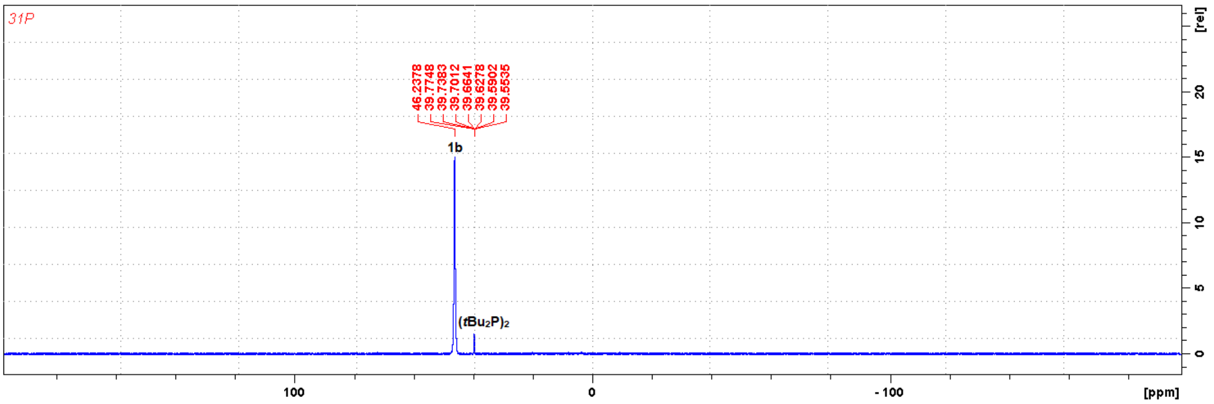

Figure S27. ^{31}P NMR (C_6D_6, 162 MHz) spectrum of **1b**.

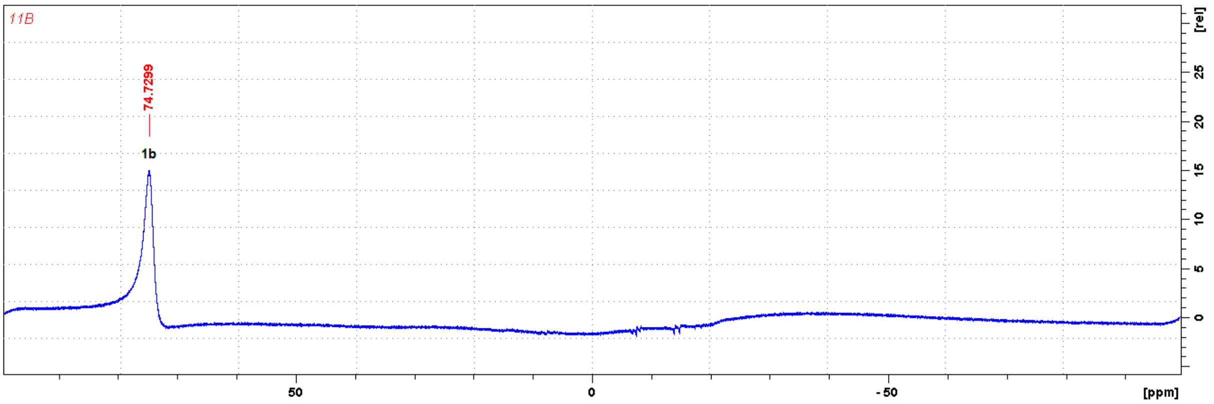

Figure S28. ^{11}B NMR (C_6D_6, 128 MHz) spectrum of **1b**.

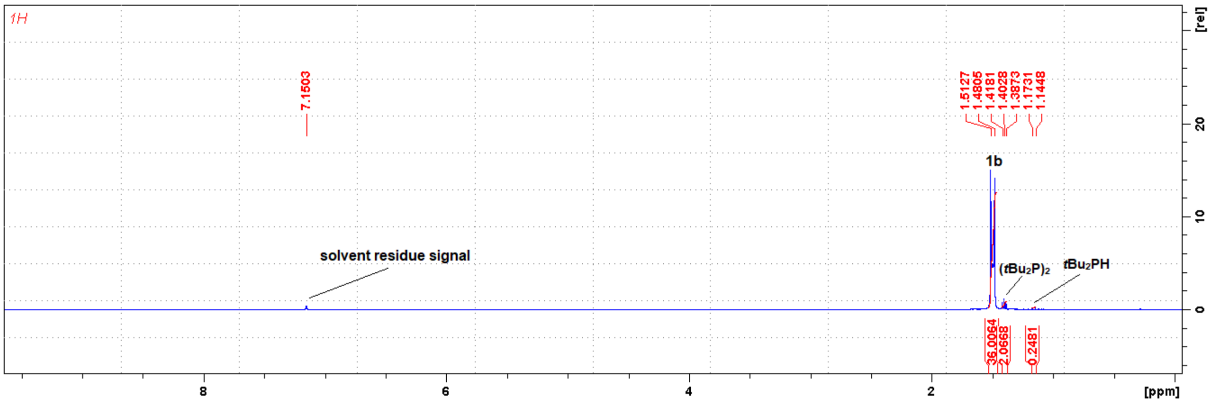

Figure S29. ^{1}H NMR (C_6D_6, 400 MHz) spectrum of **1b**.

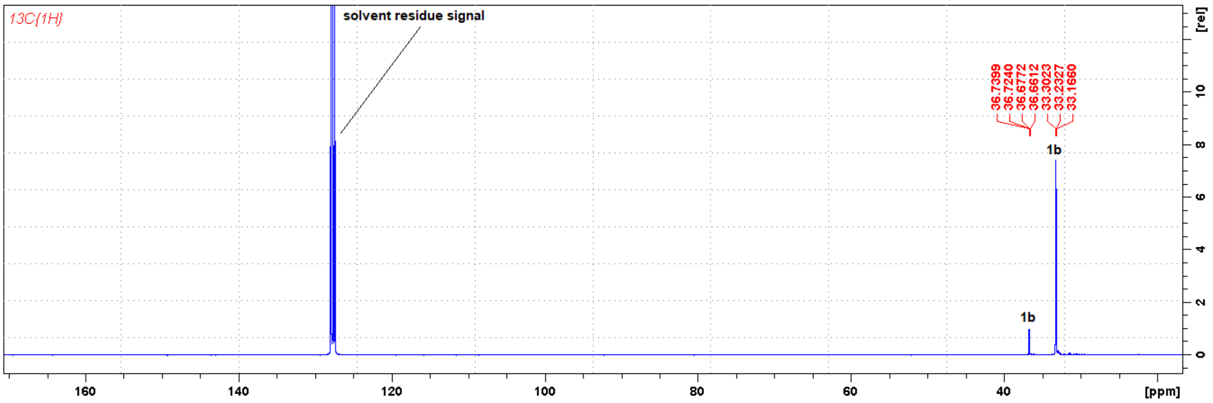

Figure S30. $^{13}C\{^{1}H\}$ NMR (C_6D_6, 100 MHz) spectrum of **1b**.

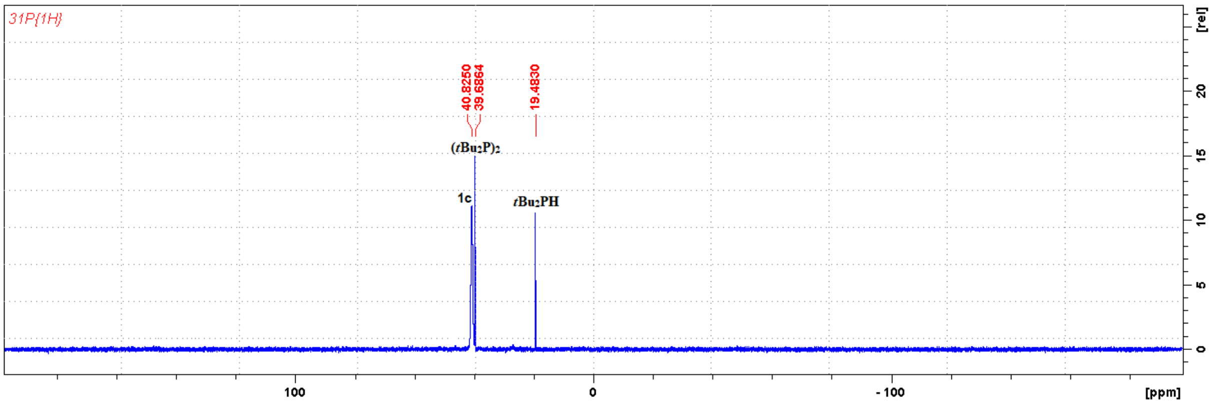

Figure S31. $^{31}P\{^{1}H\}$ NMR (C_6D_6, 162 MHz) spectrum of **1c**.

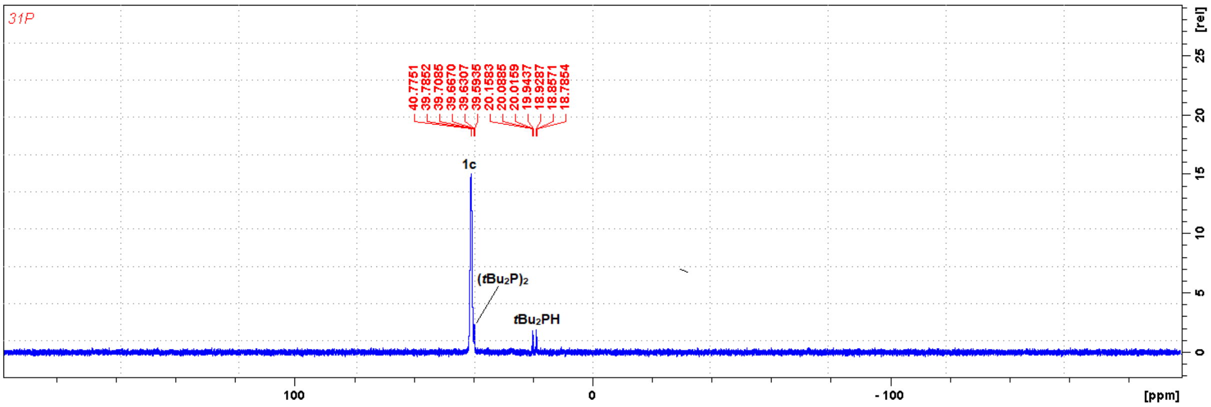

Figure S32. ^{31}P NMR (C_6D_6, 162 MHz) spectrum of **1c**.

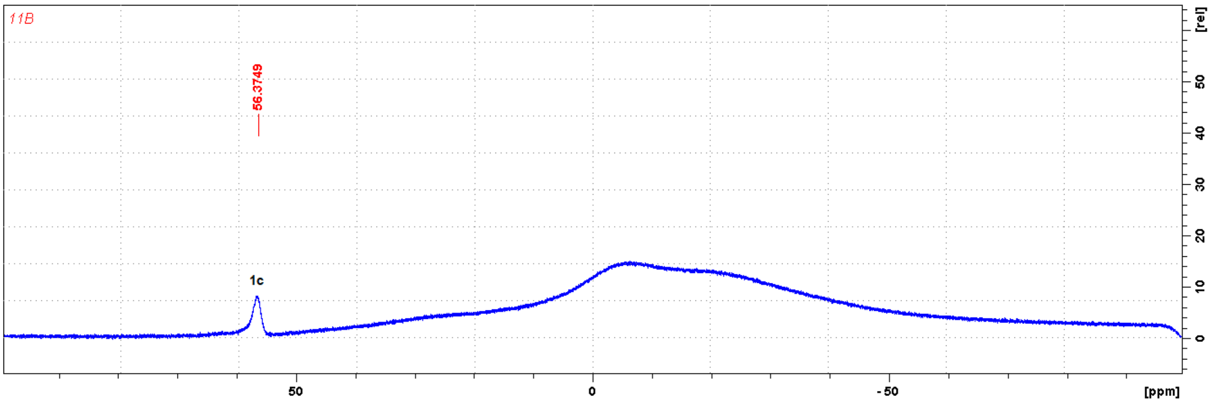

Figure S33. ^{11}B NMR (C_6D_6, 128 MHz) spectrum of **1c**.

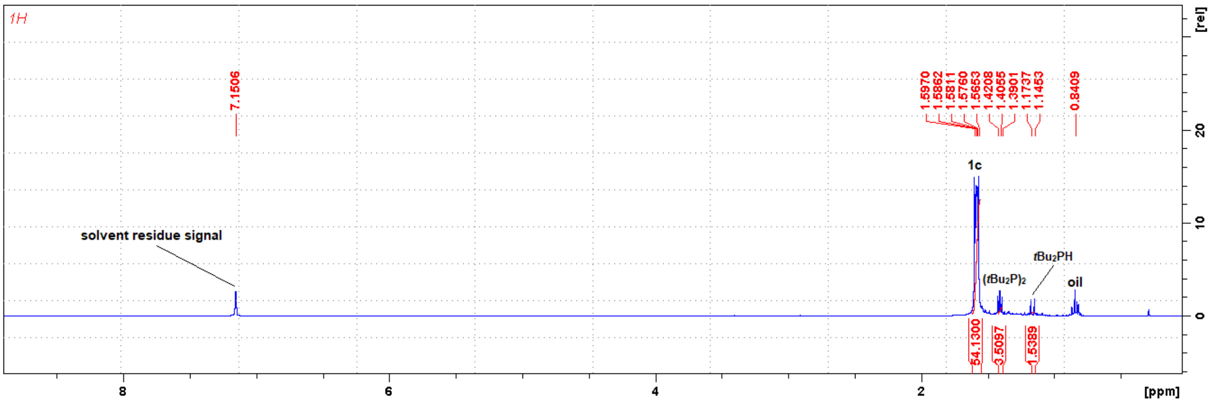

Figure S34. ^{1}H NMR (C_6D_6, 400 MHz) spectrum of **1c**.

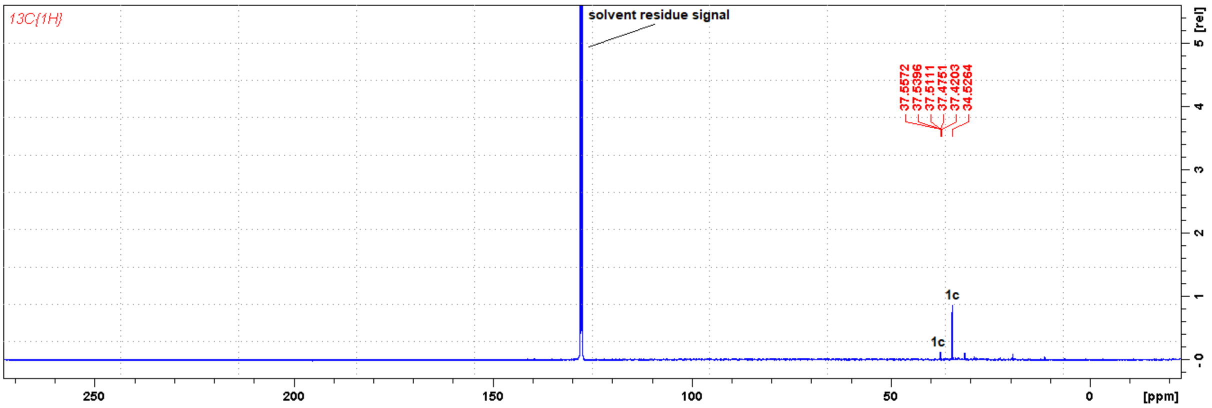

Figure S35. $^{13}C\{^1H\}$ NMR (C_6D_6, 100 MHz) spectrum of **1c**.

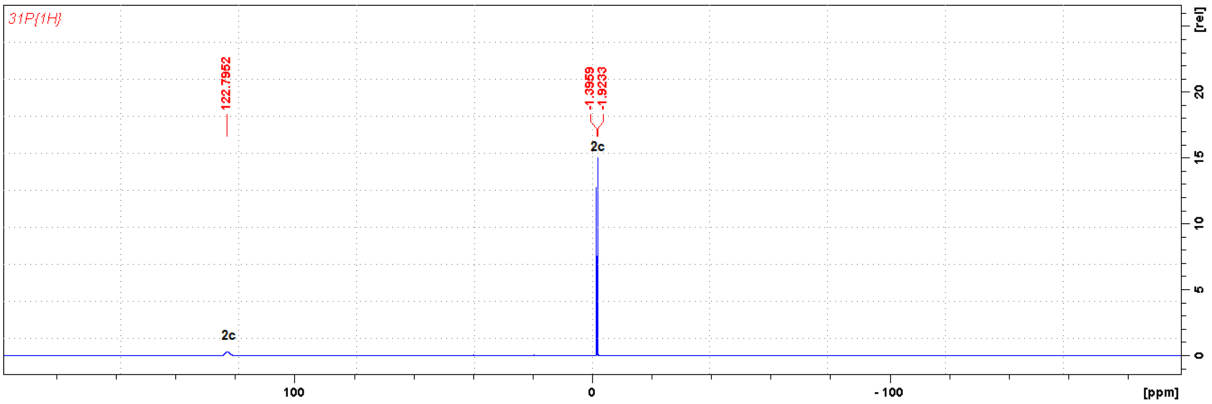

Figure S36. $^{31}P\{^1H\}$ NMR (C_6D_6, 162 MHz) spectrum of **2c**.

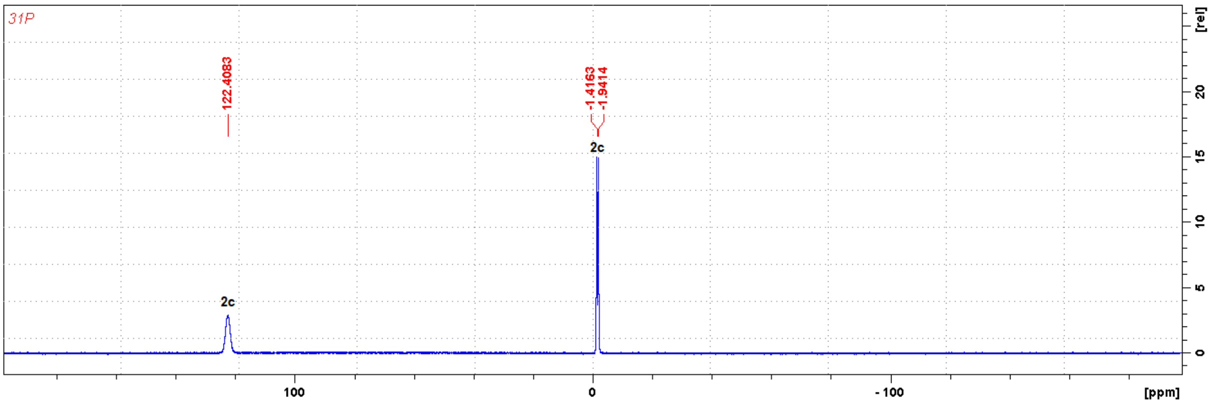

Figure S37. ^{31}P NMR (C_6D_6, 162 MHz) spectrum of **2c**.

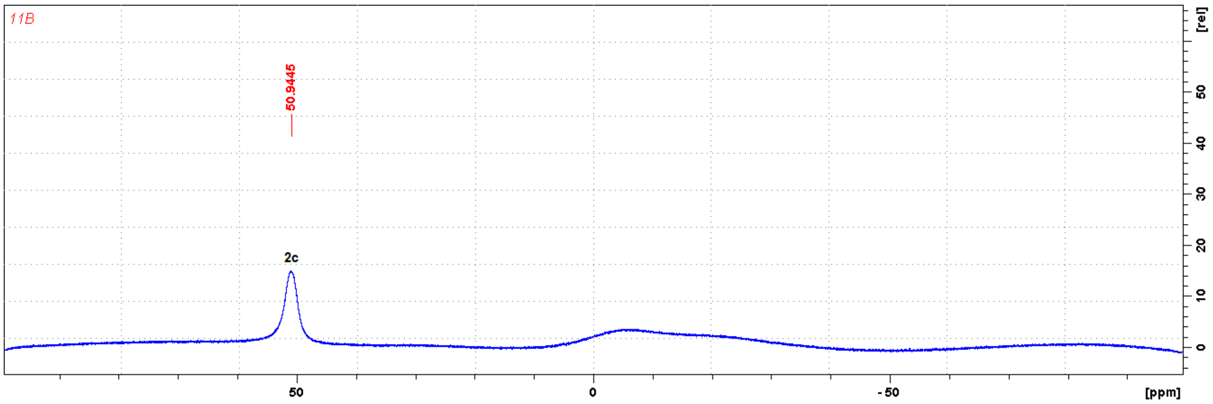

Figure S38. ^{11}B NMR (C_6D_6, 128 MHz) spectrum of **2c**.

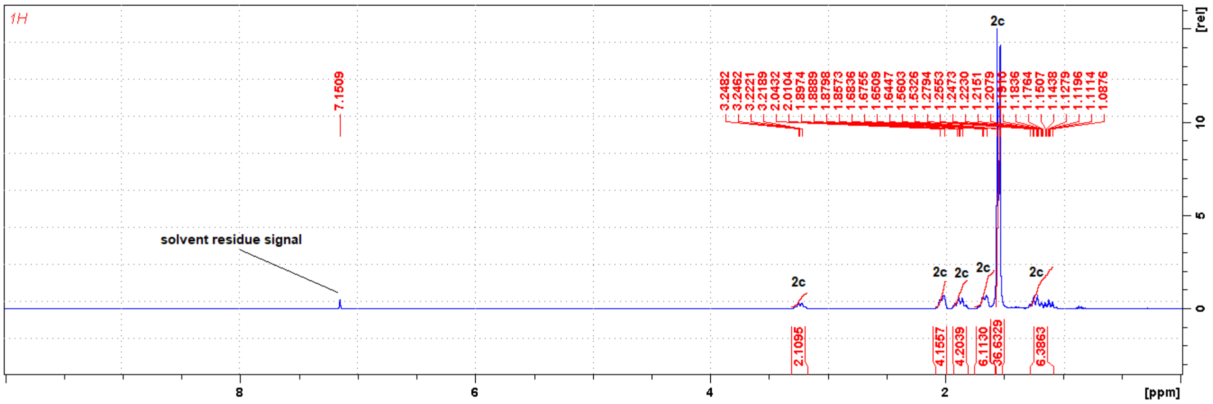

Figure S39. ^{1}H NMR (C_6D_6, 400 MHz) spectrum of **2c**.

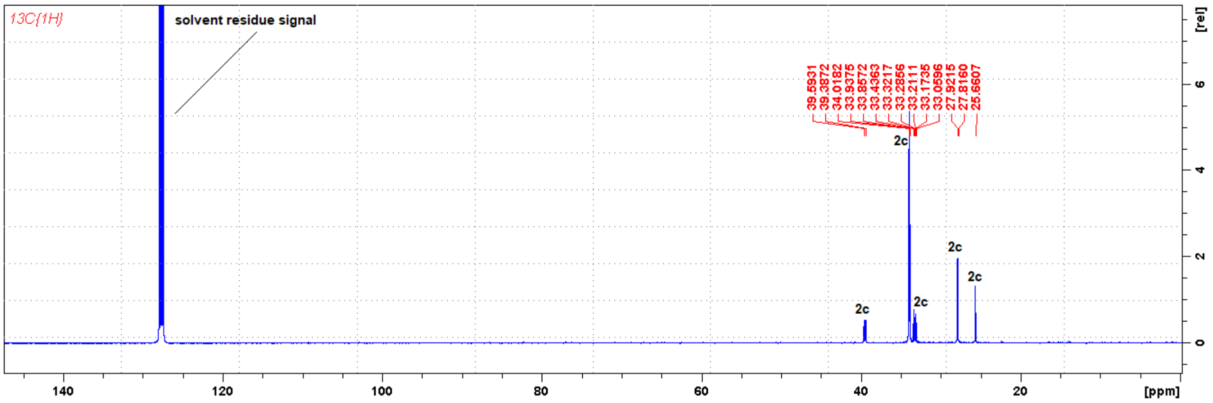

Figure S40. $^{13}C\{^{1}H\}$ NMR (C_6D_6, 100 MHz) spectrum of **2c**.

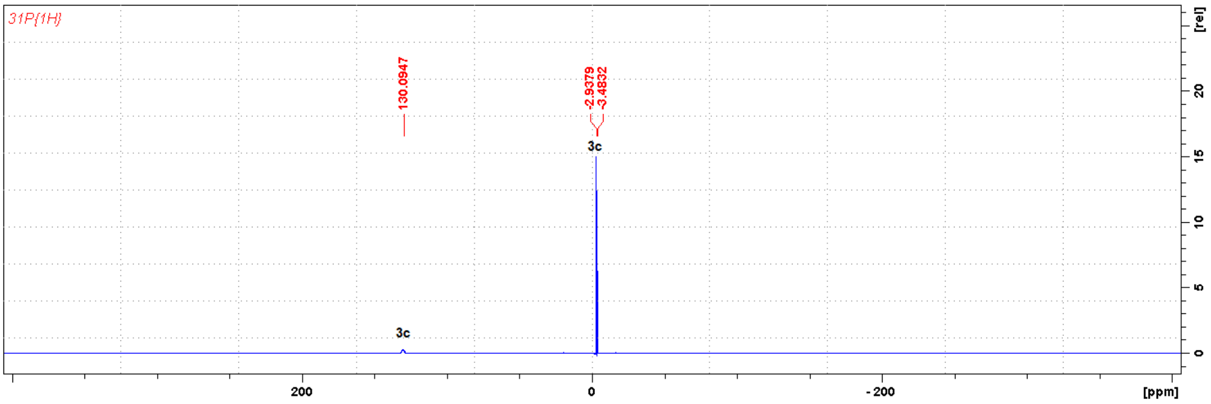

Figure S41. $^{31}P\{^{1}H\}$ NMR (C_6D_6, 162 MHz) spectrum of **3c**.

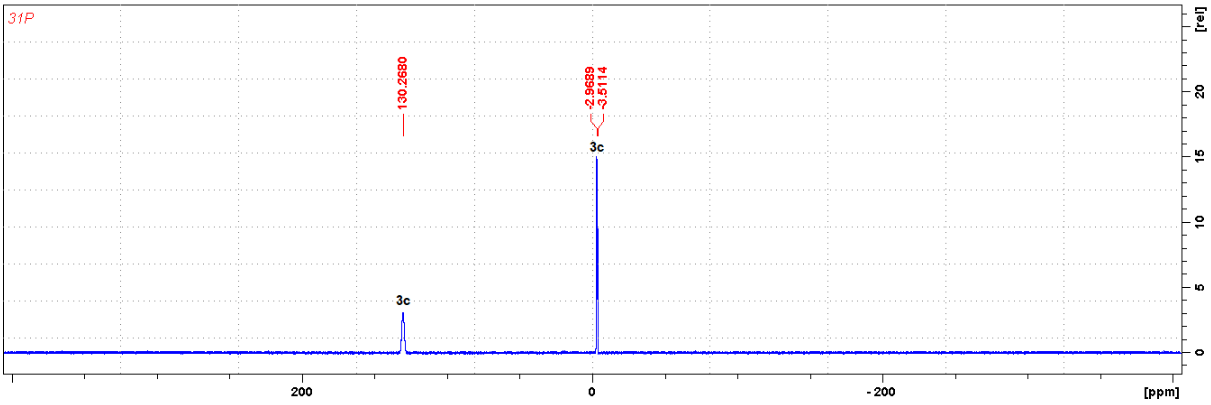

Figure S42. ^{31}P NMR (C_6D_6, 162 MHz) spectrum of **3c**.

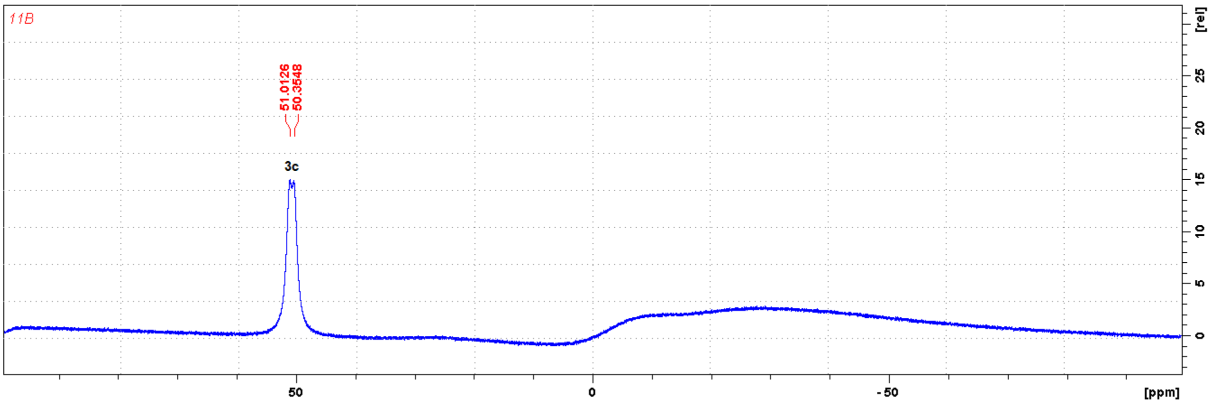

Figure S43. ^{11}B NMR (C_6D_6, 128 MHz) spectrum of **3c**.

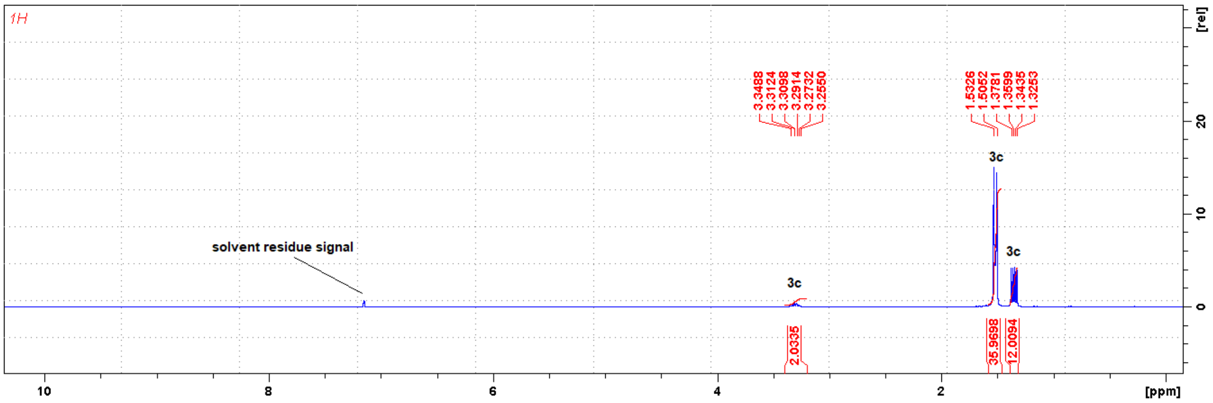

Figure S44. ^{1}H NMR (C_6D_6, 400 MHz) spectrum of **3c**.

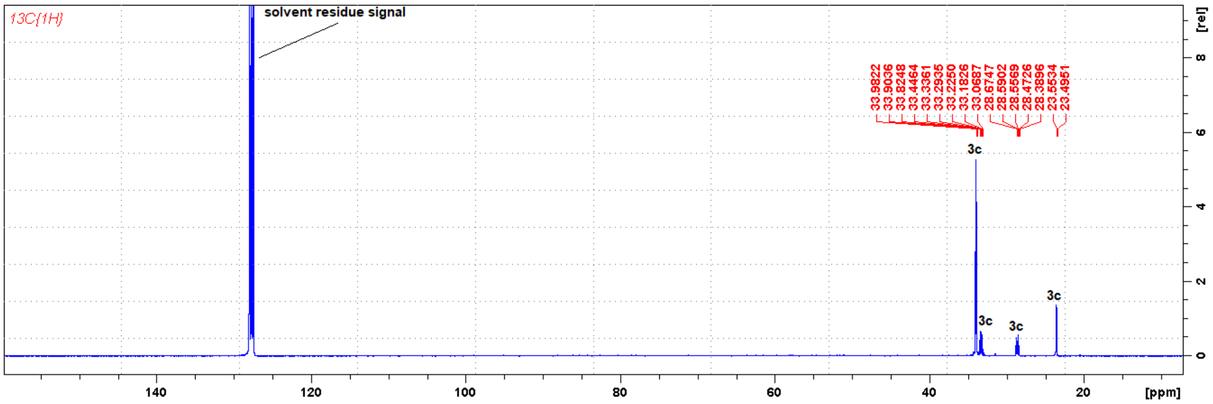

Figure S45. $^{13}C\{^{1}H\}$ NMR (C_6D_6, 100 MHz) spectrum of **3c**.

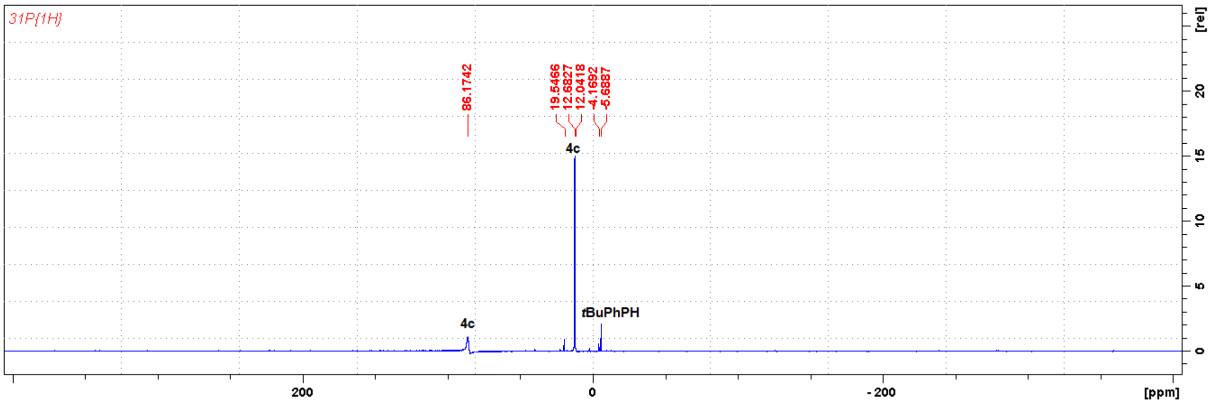

Figure S46. $^{31}P\{^{1}H\}$ NMR (C_6D_6, 162 MHz) spectrum of **4c**.

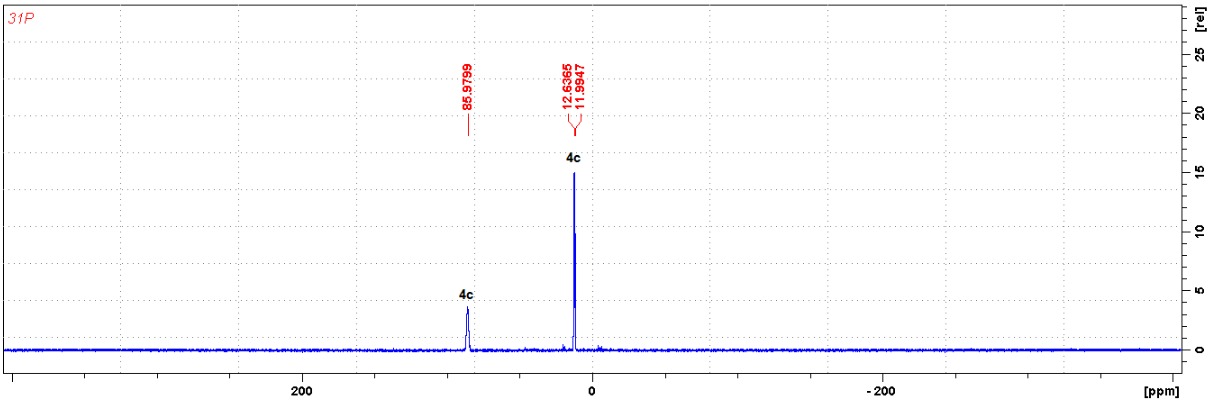

Figure S47. ^{31}P NMR (C_6D_6, 162 MHz) spectrum of **4c**.

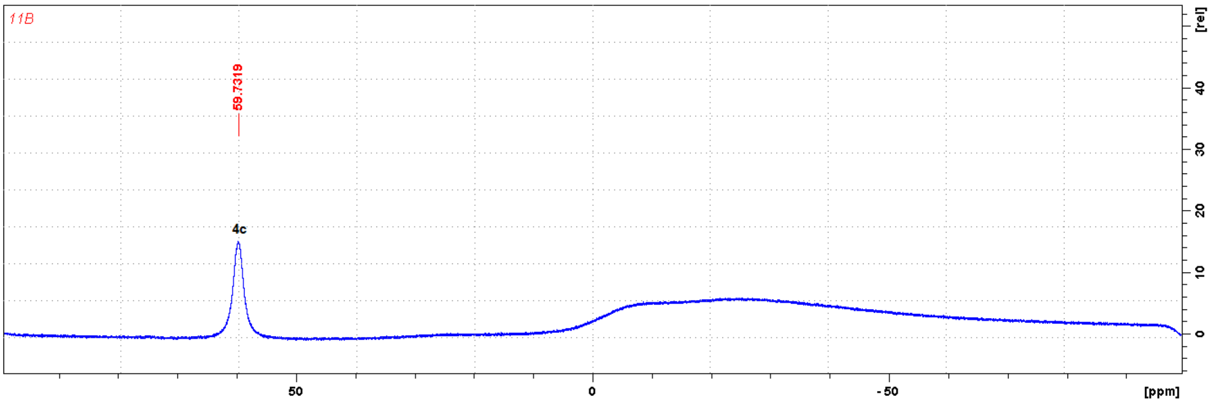

Figure S48. ^{11}B NMR (C_6D_6, 128 MHz) spectrum of **4c**.

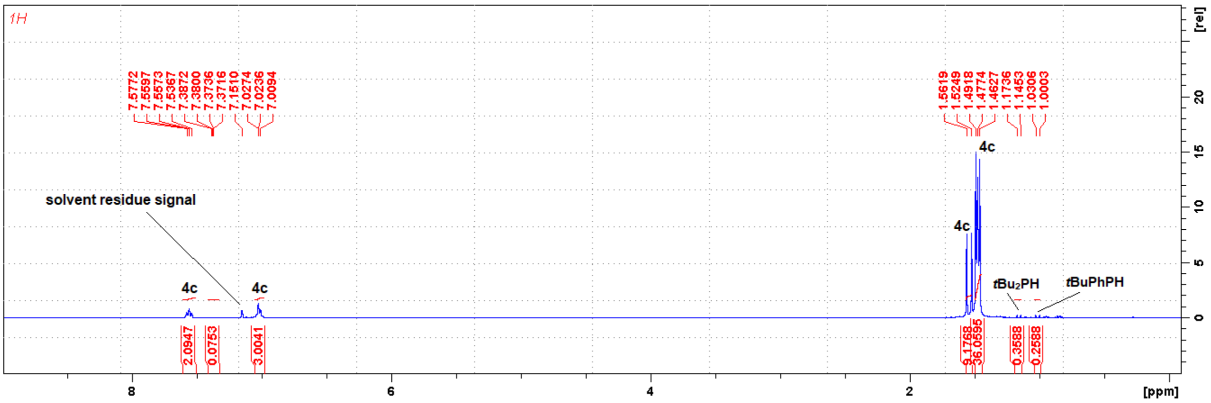

Figure S49. ^{1}H NMR (C_6D_6, 400 MHz) spectrum of **4c**.

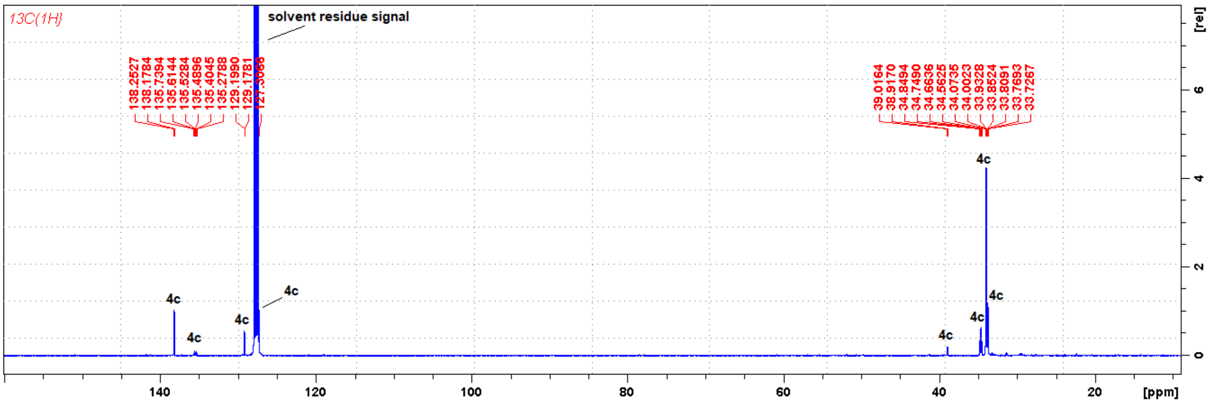

Figure S50. $^{13}C\{^{1}H\}$ NMR (C_6D_6, 100 MHz) spectrum of **4c**.

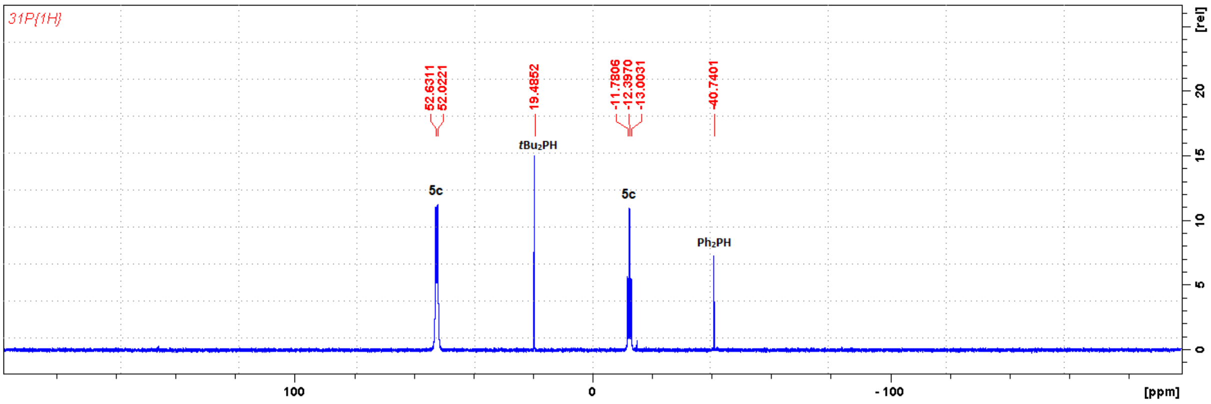

Figure S51. $^{31}P\{^{1}H\}$ NMR (C_6D_6, 162 MHz) spectrum of **5c**.

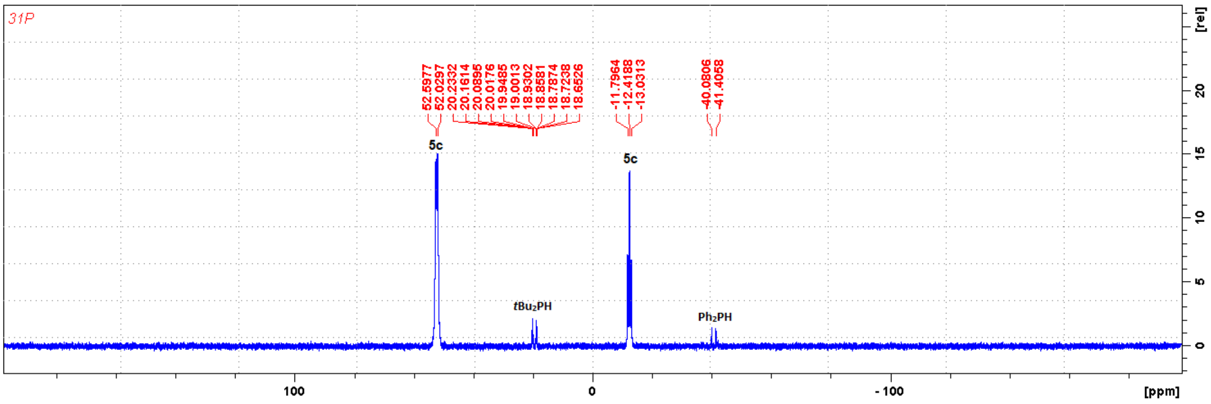

Figure S52. ^{31}P NMR (C_6D_6, 162 MHz) spectrum of **5c**.

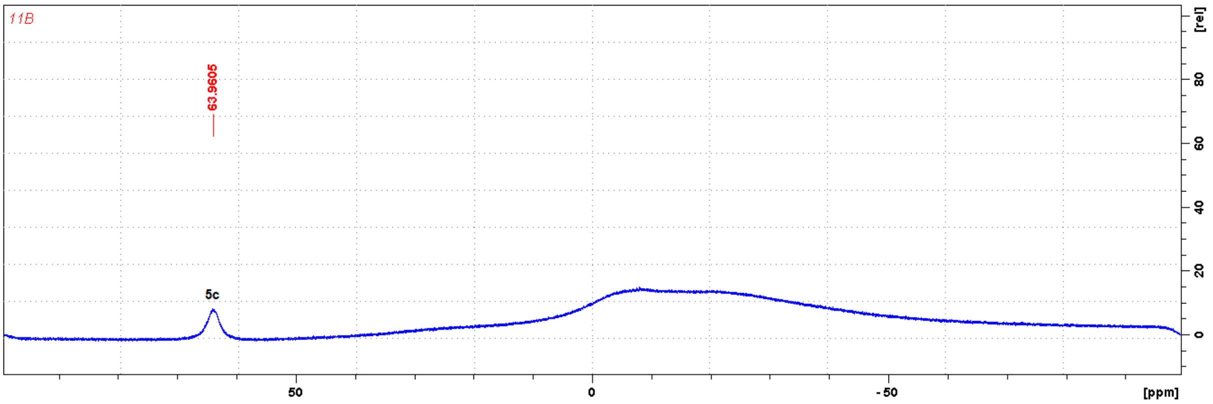

Figure S53. ^{11}B NMR (C_6D_6, 128 MHz) spectrum of **5c**.

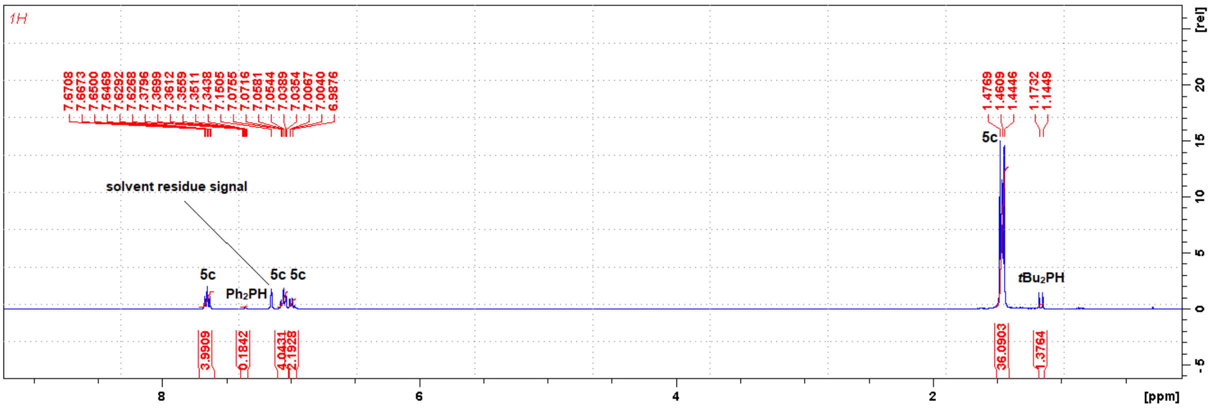

Figure S54. ^{1}H NMR (C_6D_6, 400 MHz) spectrum of **5c**.

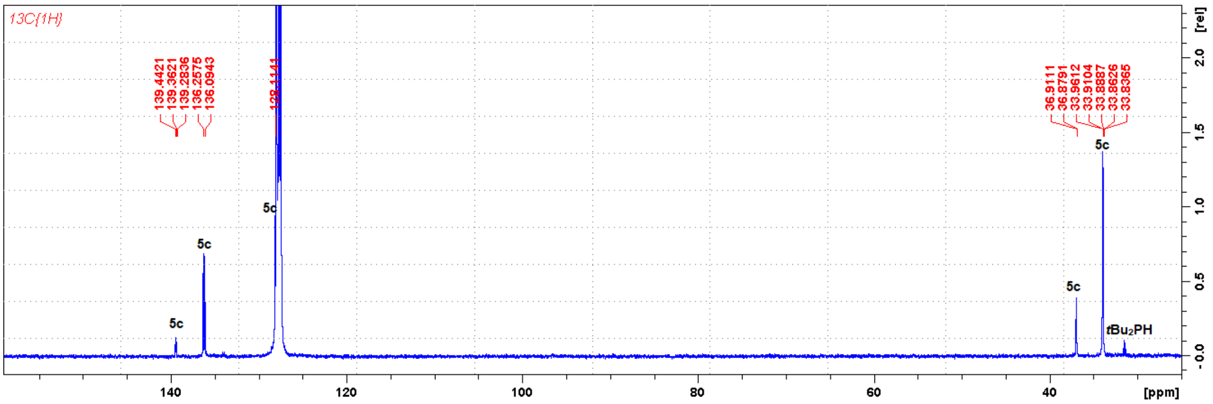

Figure S55. $^{13}C\{^{1}H\}$ NMR (C_6D_6, 100 MHz) spectrum of **5c**.

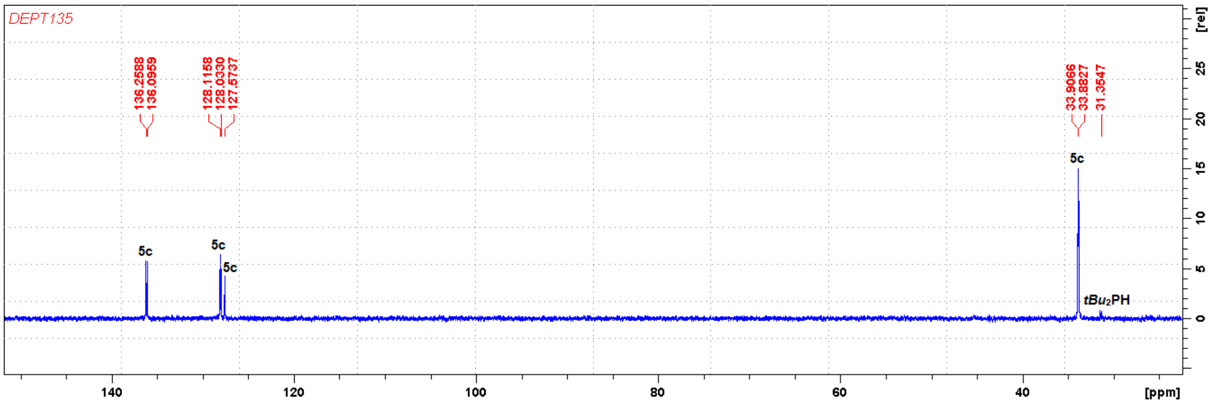

Figure S56. DEPT-135 NMR (C_6D_6, 100 MHz) of **5c**.

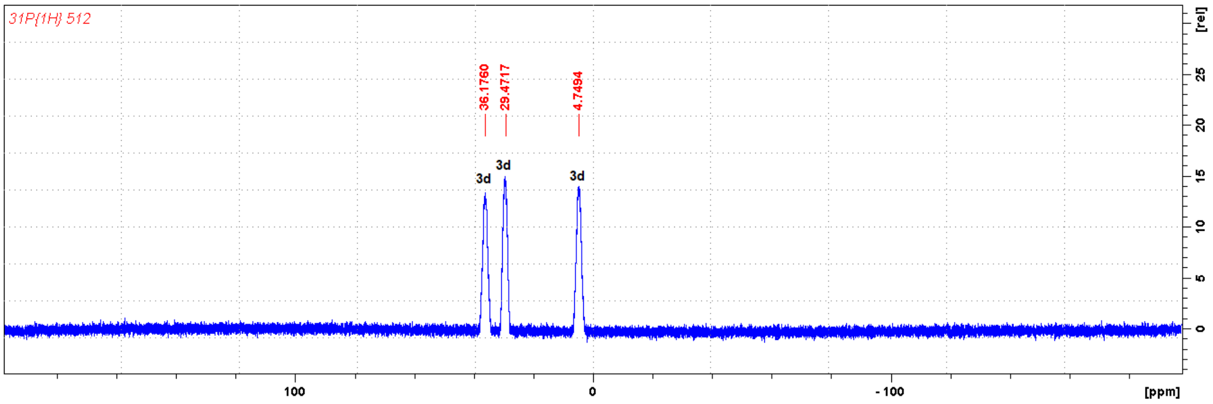

Figure S57. $^{31}P\{^{1}H\}$ NMR (C_6D_6, 162 MHz) spectrum of **3d**.

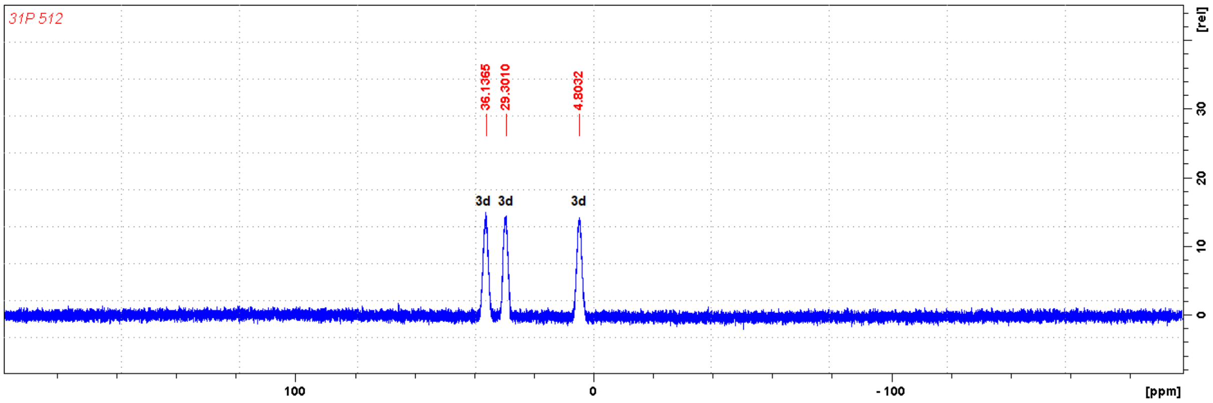

Figure S58. ^{31}P NMR (C_6D_6, 162 MHz) spectrum of **3d**.

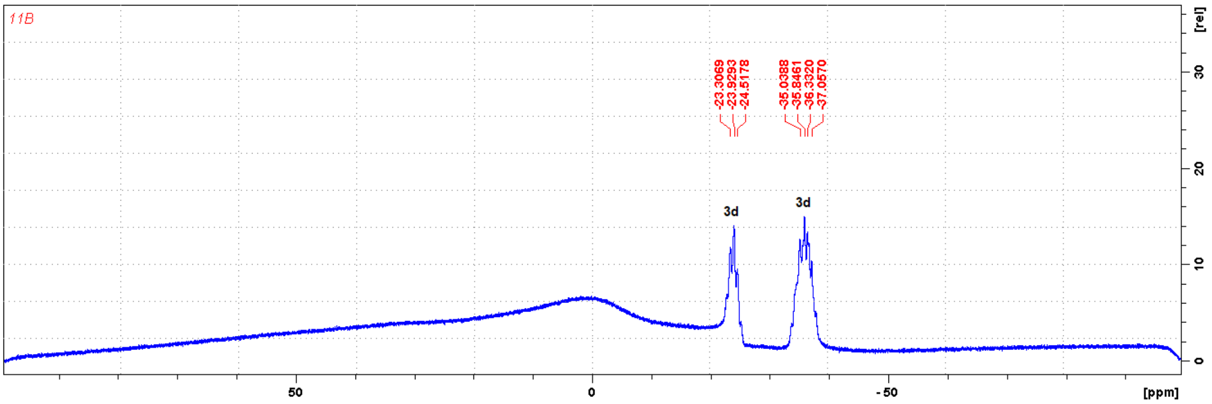

Figure S59. ^{11}B NMR (C_6D_6, 128 MHz) spectrum of **3d**.

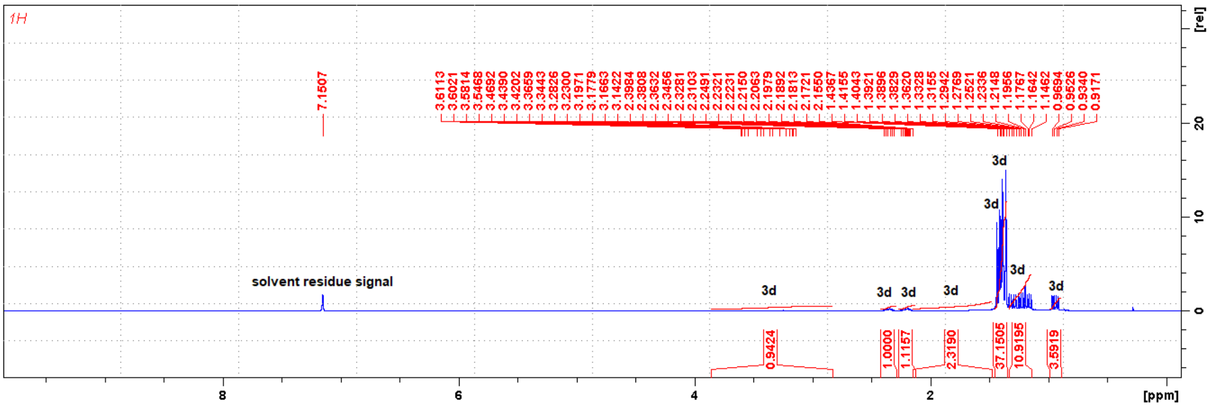

Figure S60. ^{1}H NMR (C_6D_6, 400 MHz) spectrum of **3d**.

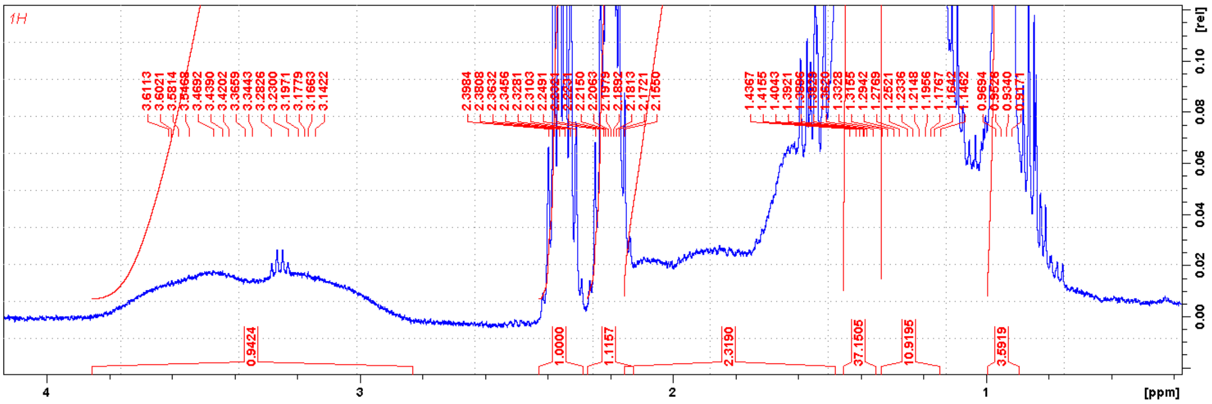

Figure S61. ^{1}H NMR (C_6D_6, 400 MHz) spectrum with selected range of **3d**.

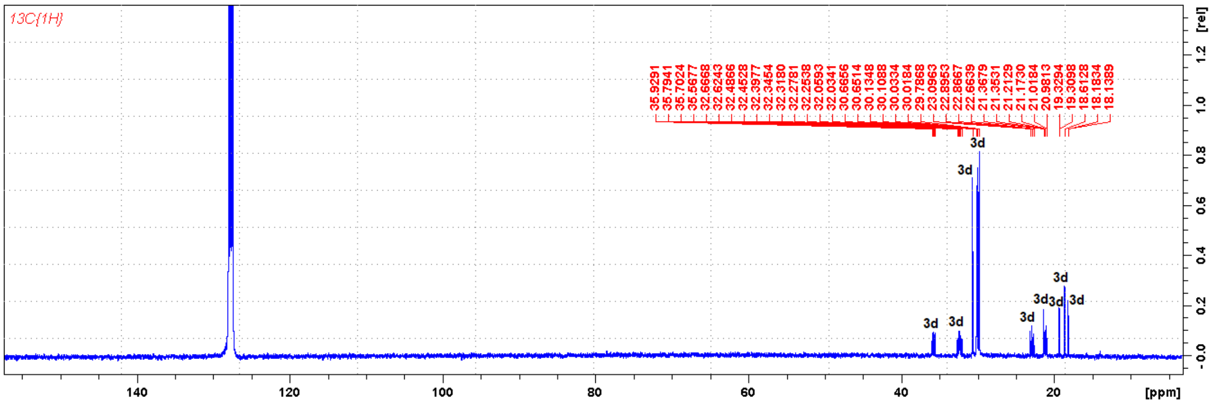

Figure S62. $^{13}C\{^{1}H\}$ NMR (C_6D_6, 100 MHz) spectrum of **3d**.

3. Computational Details

All calculations presented in the paper were performed using the Gaussian 09[4] program package. The molecular geometry of compound **1c** was optimized using density functional theory at the TPSSTPSS functional by Tao *et al.* [5] with 6-31+G(d,p) basis set. Optimization of **1c** was performed due to the presence of two different conformers in the crystal unit (resulting from an X-ray analysis of **1c**). The TPSSTPSS exchange-correlation functional has been chosen for its good overall performance for describing main-group element compounds. Adding the GD3BJ keyword that includes the D3 version of Grimme dispersion with Becke-Johnson7 damping into calculations also accounts well for long-range and dispersion interactions. NBO and NLMO analysis was performed for non-optimized structures of **2c**, **3c**, **4c**, **4c'** and **5c** derived from X-ray analysis and optimized structure of **1c** at TPSSTPSS//6-31+G(d,p) level of theory by applying the NBO 3.1[6] module built-in Gaussian 09.

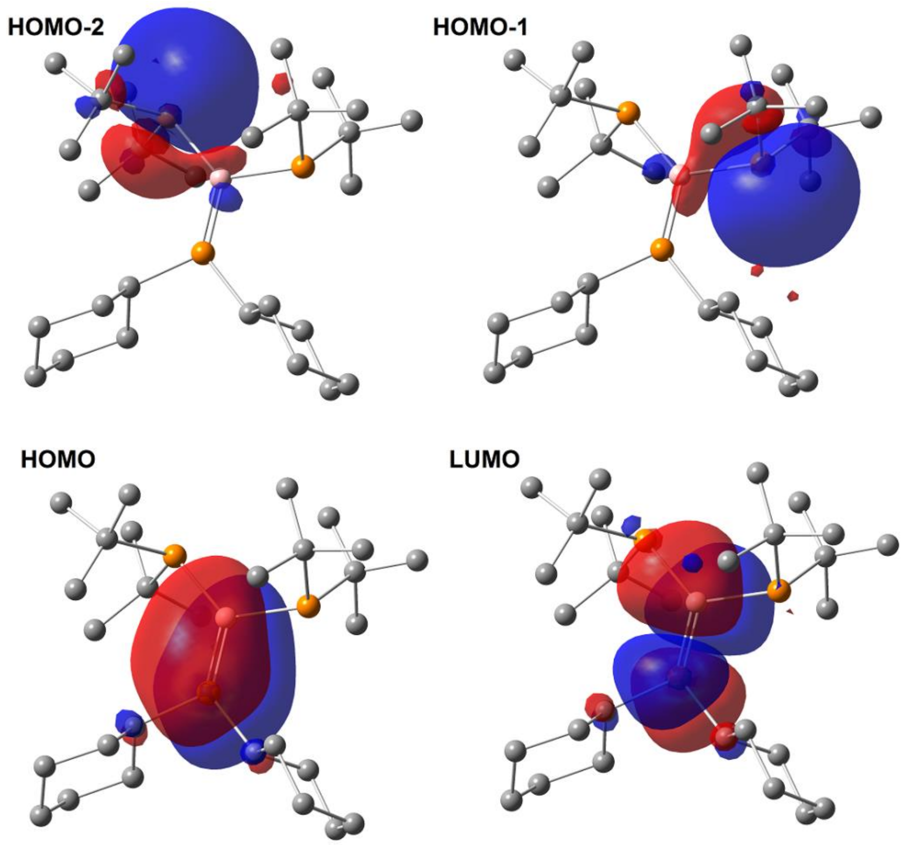

Figure S63. Graphical representation of the NBOs of **2c**.

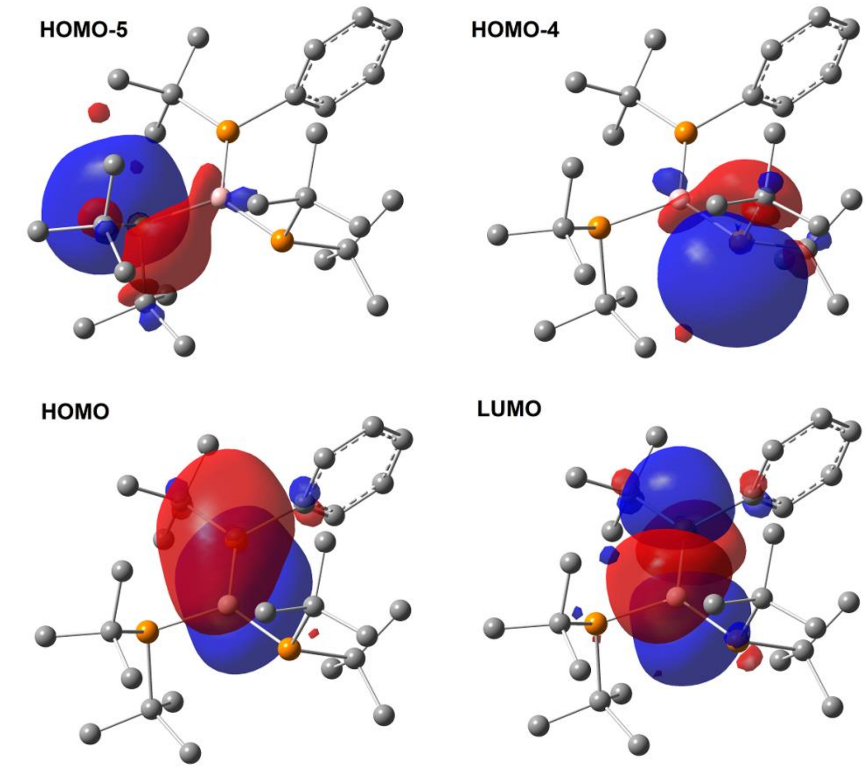

Figure S64. Graphical representation of the NBOs of **4c**.

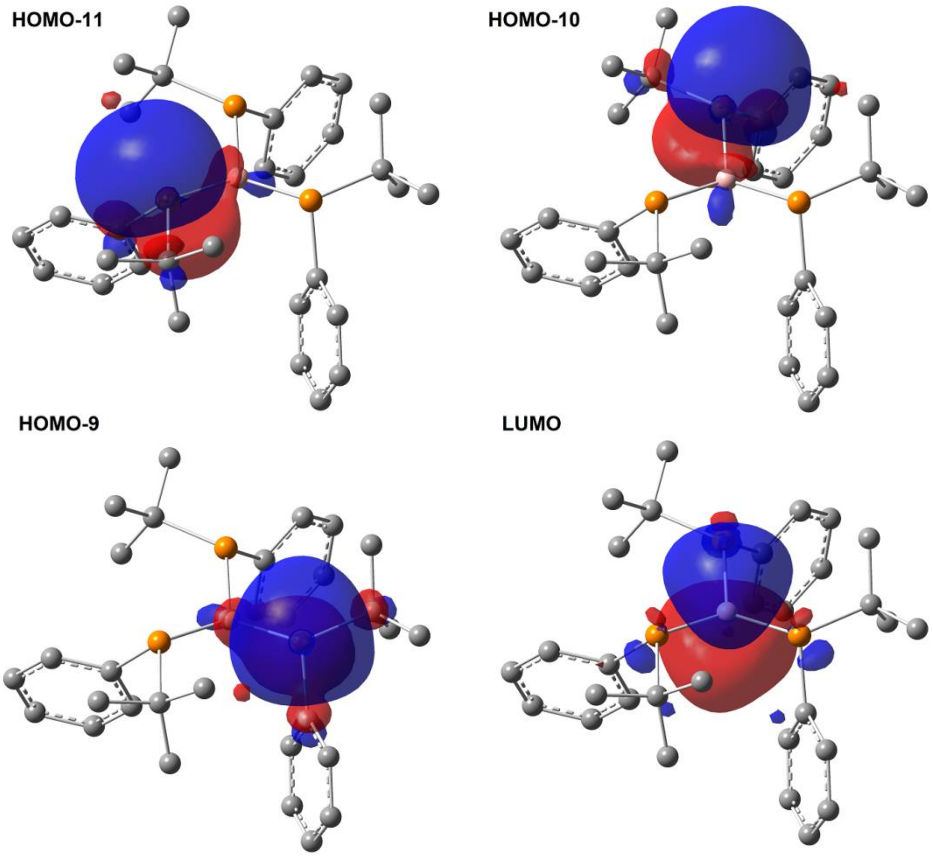

Figure S65. Graphical representation of the NBOs of **4c'**.

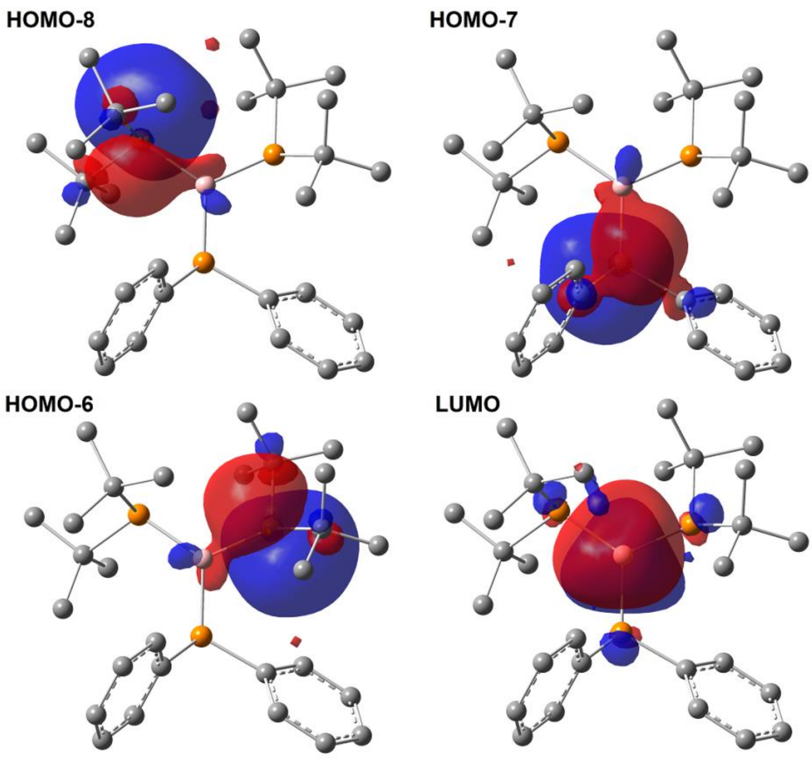

Figure S66. Graphical representation of the NBOs of **5c**.

Table S4. Cartesian coordinates of optimized structure **1c**.

P	-0.74968300	1.74250500	0.30723600
C	-2.42930700	1.82485700	1.22239500
C	-2.15906300	1.17874800	2.60360100
H	-1.88689800	0.12283900	2.49805800
H	-3.07408900	1.24114900	3.21272100
H	-1.35419900	1.70118700	3.13581600
C	-3.63088800	1.11245400	0.57504000
H	-3.85596400	1.49395500	-0.42684900
H	-4.52626300	1.26121000	1.20035600
H	-3.43190900	0.03750400	0.51282500
C	-2.77568600	3.31205200	1.46509900
H	-1.93475700	3.85044300	1.92055300
H	-3.63118500	3.36727400	2.15521000
H	-3.06389900	3.83023100	0.54306500
C	-0.74585100	2.86402300	-1.25835700
C	-2.07674600	2.84812900	-2.03801900
H	-2.32720900	1.84394700	-2.39165900
H	-1.97341100	3.50145100	-2.91904200
H	-2.91303400	3.23004100	-1.44286300
C	-0.42251100	4.32319300	-0.85813400
H	-1.19102400	4.74937100	-0.20542800
H	-0.36952700	4.94366900	-1.76631000

H	0.54059700	4.39375800	-0.34069900
C	0.36927400	2.35425700	-2.18908500
H	1.33564400	2.34680500	-1.67790400
H	0.44720100	3.02063900	-3.06198900
H	0.16026500	1.33753000	-2.54217200
B	-0.00057900	0.00052100	-0.00041500
P	-1.13500400	-1.51986600	0.30509500
C	-0.36776900	-3.01433600	1.22264500
C	0.05606500	-2.45479100	2.60303900
H	0.83316700	-1.69004000	2.49624600
H	0.46081800	-3.27698600	3.21323900
H	-0.79925900	-2.01937200	3.13480300
C	0.84994100	-3.70054500	0.57738300
H	0.63267100	-4.08625800	-0.42459600
H	1.16703600	-4.55051100	1.20336300
H	1.68242900	-2.99191900	0.51590000
C	-1.48318900	-4.05701300	1.46681200
H	-2.37084300	-3.59570500	1.91812600
H	-1.10507400	-4.82257400	2.16116000
H	-1.78575900	-4.56997400	0.54637700
C	-2.10697200	-2.07918600	-1.26100900
C	-1.42780400	-3.22515800	-2.03888600
H	-0.43232900	-2.94151500	-2.39203600
H	-2.04469000	-3.46249000	-2.92027000
H	-1.34199200	-4.13998900	-1.44297100
C	-3.53245900	-2.52826900	-0.86074100
H	-3.51744000	-3.40537100	-0.20602000
H	-4.09555200	-2.79487500	-1.76873700
H	-4.07572400	-1.72837800	-0.34563800
C	-2.22359300	-0.86020600	-2.19396200
H	-2.70434300	-0.02008900	-1.68563600
H	-2.83688300	-1.12910400	-3.06786600
H	-1.23879300	-0.53074800	-2.54569500
P	1.88338800	-0.22115600	0.30219000
C	2.79713800	1.19055900	1.21806200
C	2.10362700	1.27779600	2.60004400
H	1.05236700	1.56805300	2.49602200
H	2.61451500	2.03962700	3.20897000
H	2.15617800	0.31940200	3.13170900
C	2.78286900	2.58871900	0.57347600
H	3.22457500	2.59406300	-0.42899300
H	3.36208700	3.28692100	1.19946500
H	1.75353200	2.95719000	0.51340700
C	4.25823000	0.74542800	1.45962200
H	4.30357000	-0.25373200	1.91134900

H	4.73347200	1.45594300	2.15279900
H	4.85208400	0.73946900	0.53810600
C	2.85198700	-0.78567500	-1.26381400
C	3.50515100	0.37549700	-2.04130800
H	2.76198500	1.09515700	-2.39634600
H	4.02169300	-0.03979600	-2.92131400
H	4.25217800	0.90802800	-1.44335400
C	3.95313700	-1.79607400	-0.86328700
H	4.70573000	-1.34484500	-0.20896300
H	4.46502600	-2.15139700	-1.77120000
H	3.53125900	-2.66578200	-0.34764000
C	1.85438800	-1.49596400	-2.19649000
H	1.36466300	-2.33016400	-1.68709300
H	2.39427400	-1.89549200	-3.06885100
H	1.07861900	-0.80701200	-2.55076100

5. References

(1) Dolomanov, O. V; Bourhis, L. J.; Gildea, R. J.; Howard, J. A. K.; Puschmann, H. *J. Appl. Crystallogr.* **2009**, *42*, 339–341.

(2) Sheldrick, G. M. *Acta Cryst. A* **2015**, *71* (1), 3–8.

(3) Sheldrick, G. M. *Acta Cryst. C* **2015**, *71*, 3–8.

(4) Frisch, M. J.; Trucks, G. W.; Schlegel, H. B.; Scuseria, G. E.; Robb, M. A.; Cheeseman, J. R.; Scalmani, G.; Barone, V.; Petersson, G. A.; Nakatsuji, H.; Li, X.; Caricato, M.; Marenich, A.; Bloino, J.; Janesko, B. G.; Gomperts, R.; Mennucci, B.; Hratchian, H. P.; Ortritz, J. V.; Izmaylov, A. F.; Sonnenberg, J. L.; Williams-Young, D.; Ding, F.; Lipparini, F.; Egidi, F.; Goings, J.; Peng, B.; Petrone, A.; Henderson, T.; Ranasinghe, D.; Zakrzewski, V. G.; Gao, J.; Rega, N.; Zheng, G.; Liang, W.; Hada, M.; Ehara, M.; Toyota, K.; Fukuda, R.; Hasegawa, J.; Ishida, M.; Nakajima, T.; Honda, Y.; Kitao, O.; Nakai, H.; Vreven, T.; Throsse, K.; Foresman, J. B.; Fox, D. J. Gaussian, Inc.: Wallingford CT 2016.

(5) Tao, J.; Perdew, J. P.; Staroverov, V. N.; Scuseria, G. E. *Phys. Rev. Lett.* **2003**, *91* (14), 146401.

(6) Glendening, E. D.; Reed, A. E.; Carpenter, J. E.; Weinhold, F. .
